# LncRNAs as nodes for the cross-talk between autophagy and Wnt signaling in pancreatic cancer drug resistance

**DOI:** 10.7150/ijbs.91832

**Published:** 2024-04-29

**Authors:** Yuhan Huang, Rui Zhang, Hao Lyu, Shuai Xiao, Dong Guo, Xing-Zhen Chen, Cefan Zhou, Jingfeng Tang

**Affiliations:** 1 National "111" Center for Cellular Regulation and Molecular Pharmaceutics, Key Laboratory of Fermentation Engineering (Ministry of Education), Cooperative Innovation Center of Industrial Fermentation (Ministry of Education & Hubei Province), Hubei Key Laboratory of Industrial Microbiology, Hubei University of Technology, Wuhan, China, 430068.; 2 Membrane Protein Disease Research Group, Department of Physiology, Faculty of Medicine and Dentistry, University of Alberta, Edmonton, AB, Canada, T6G2R3.

**Keywords:** autophagy, drug resistance, lncRNA, pancreatic cancer, Wnt/β-catenin signaling

## Abstract

Pancreatic cancer is a malignancy with high mortality. In addition to the few symptoms until the disease reaches an advanced stage, the high fatality rate is attributed to its rapid development, drug resistance and lack of appropriate treatment. In the selection and research of therapeutic drugs, gemcitabine is the first-line drug for pancreatic cancer. Solving the problem of gemcitabine resistance in pancreatic cancer will contribute to the progress of pancreatic cancer treatment. Long non coding RNAs (lncRNAs), which are RNA transcripts longer than 200 nucleotides, play vital roles in cellular physiological metabolic activities. Currently, our group and others have found that some lncRNAs are aberrantly expressed in pancreatic cancer cells, which can regulate the process of cancer through autophagy and Wnt/β-catenin pathways simultaneously and affect the sensitivity of cancer cells to therapeutic drugs. This review presents an overview of the recent evidence concerning the node of lncRNA for the cross-talk between autophagy and Wnt/β-catenin signaling in pancreatic cancer, together with the practicability of lncRNAs and the core regulatory factors as targets in therapeutic resistance.

## Introduction

Pancreatic cancer (PC) is one of the deadliest cancers. Ductal differentiation is a common phenomenon in PC (>90%) 1, and invasive ductal adenocarcinoma is a cardinal type, accounting for the majority of pancreatic tumors (>85%) 2. Neuroendocrine tumors and acinar carcinomas are infrequent while more infrequent tumors include colloid carcinomas, pancreatoblastomas and solid-pseudopapillary neoplasms 3. Now, pancreatic ductal adenocarcinoma (PDAC) has become a synonym for PC and can directly refer to PC 3. According to the American Cancer Society, in 2023, there will be 64,050 new cancer cases of PC in the United States, accounting for 3.27% of the total cases (1,958,310), and 50,550 deaths, accounting for 8.29% of the total number (609,820) 4. Meanwhile, PC currently has the lowest 5-year relative survival rate of all cancers (12%) 4. This is associated with its poor prognosis due to factors such as low rate of early detection, rapid progression, development of drug resistance and lack of appropriate treatment. After long-term clinical treatment research, it has been established that the treatment of PC is mainly based on surgical resection, supplemented by chemotherapy 5. Gemcitabine is the first-line drug for the treatment of advanced PC 6, and it has shown better efficacy in combination with capecitabine 7 and albumin-bound paclitaxel 8. In addition, combination chemotherapy with fluorouracil, leucovorin, irinotecan, and oxaliplatin (FOLFIRINOX) or nanoliposome irinotecan plus fluorouracil and leucovorin has been proposed in recent years, and patients treated with combination therapy have longer overall survival than those treated with gemcitabine alone 9, 10. However, even though adjuvant therapy has been improved, the mortality rate of PC patients has remained stubbornly high, which is associated to chemotherapy resistance. Admittedly, PC cells can develop resistance to gemcitabine in a variety of ways 11, and most of the research on the chemical resistance of advanced PC has focused on gemcitabine, while the research on other drugs is still in its infancy 12. Therefore, there is an urgent demand to elucidate the mechanism of gemcitabine resistance in PC cells for the treatment.

Long non coding RNAs (lncRNAs) are transcripts larger than 200 nucleotides with no or limited protein-coding potential. More than 68% of the genes expressed in the human transcriptome are transcribed into lncRNAs 13. LncRNAs can be involved in regulating various physiological and pathological cell activities. In the nucleus, lncRNAs act as enhancers, decoys, scaffolds or guides to directly interact with DNA or chromatin regulatory factors, such as transcription factors and RNA-binding proteins, to control gene expression. Whereas in the cytoplasm, lncRNAs can competitively bind with miRNA to regulate mRNA stability and translation or recruit cofactors and influence the activity of related enzymes to regulate the transcription and translation process 14-16. Nowadays, lncRNAs have attracted more and more attention, and there is sufficient evidence that lncRNA is related to multiple diseases, especially cancer. In cancer cells, abnormally expressed lncRNA has been viewed as a classical oncogene or tumor suppressor gene, with variation in lncRNA expression species, amount of expression and even efficacy in different cancers and at different times of progression 17, 18. In our previous study, we found that in PC, there are lncRNAs simultaneously regulating Wnt/β-catenin and autophagy, which can lead to gemcitabine resistance, and lncRNAs that can be used as biomarkers for prognosis analysis were also screened through data analysis 19, 20. Therefore, we believe that lncRNAs have the potential to regulate PC progression and lead to drug resistance from both Wnt/β-catenin and autophagy pathways.

In this review, we collect lncRNAs related to Wnt/β-catenin and autophagy pathways in PC, discuss their roles in processes regulating cell metabolism and effects on chemoresistance, from which we identify the shared pathways with core factors to provide new targets and research directions for PC prevention and treatment.

### LncRNAs regulate Wnt/β-catenin signaling pathway in PC

Wnt/β-catenin signaling pathway is of vital importance in regulating various physiological activities of cells, including tissue homeostasis, cell proliferation, cell differentiation and cell death 21-23. The canonical Wnt/β-catenin pathway is activated by the binding of endocrine or paracrine Wnt ligands to Frizzled (FZD) and low density lipoprotein receptor-related protein (LRP) family membrane receptors on the cell surface. In the absence of Wnt ligand, β-catenin is captured by a complex consisting of adenomatous polyposis coli (APC), axis inhibitor (AXIN), casein kinase 1 (CK1) and glycogen synthase kinase-3 beta (GSK‑3β), resulting in degradation of β-catenin. Upon Wnt activation, the complex is recruited to the plasma membrane through interaction with the FZD, thus losing its ability to degrade β-catenin. After that, β-catenin would translocate from cytoplasm to nucleus and interact with the transcriptional response element TCF/LEF (T-cell factor/lymphoid enhancer-binding factor) to activate the transcription of target genes 24. Wnt/β-catenin signaling regulates PC from multiple perspectives including initiation, progression, propagation and treatment resistance 25, 26. In addition, the abnormal activation of Wnt promotes the immunosuppression of PC 27, promotes the development and differentiation of pancreatic cancer stem cells (PaCSCs) 28, and is associated with poor prognosis 29.

**Figure 1:**
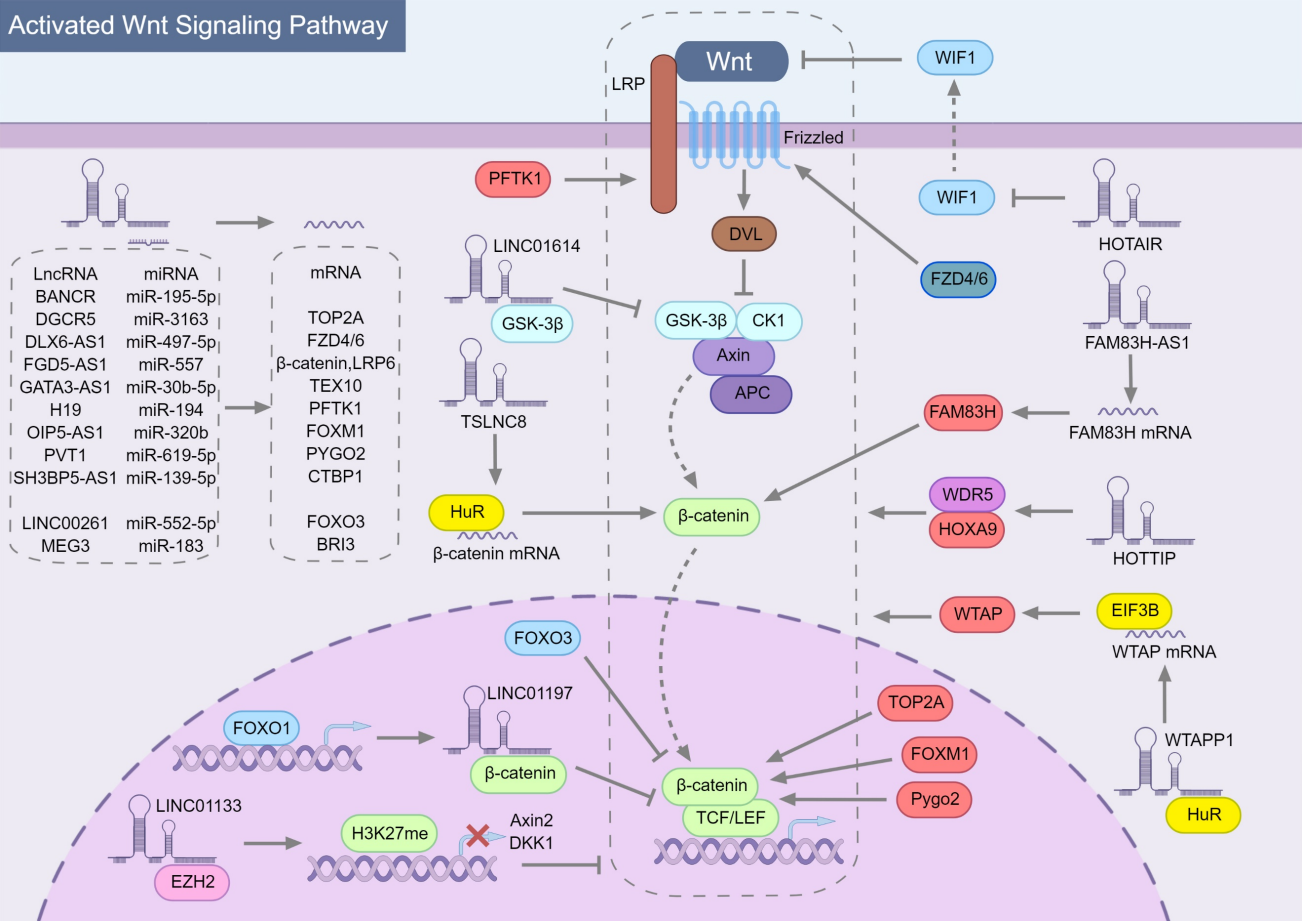
** The role of lncRNAs in modulating the Wnt/β-catenin signaling pathway in pancreatic cancer.** Most lncRNAs indirectly regulate Wnt/β-catenin signaling pathway through sponging miRNA, among which the top half of lncRNAs have an activation effect and the bottom two have an inhibitory effect. The targets of miRNAs are directly the components of the Wnt/β-catenin pathway, while other target proteins regulate the components of the Wnt/β-catenin pathway. Among them, factors that can activate Wnt/β-catenin are marked in red, and those that inhibit Wnt/β-catenin are marked in blue. In addition, there are also lncRNAs (such as LINC01614 and LINC01197) that directly bind to Wnt/β-catenin pathway proteins and inhibit their physiological activity. Specifically, HuR can stabilize RNA, including mRNA and lncRNA. The combination of TSLNC8, HuR and β-catenin mRNA can promote β-catenin translation and HuR can stabilize WTAPP1, which enhances WTAP translation by collecting EIF3B into WTAP mRNA and finally regulates Wnt/β-catenin pathway.

In general, lncRNAs can regulate the Wnt/β-catenin pathway in multiple ways (Fig. 1), but mainly by spongating miRNA to stabilize mRNA and then up-regulate the expression of the corresponding protein. Among these studies shown in Table 1 (23 articles), nearly half (11 articles) described that lncRNAs could act as competitive endogenous RNAs (ceRNAs) to competitively adsorb miRNAs, resulting in loss or attenuation of miRNA function and promoting the expression of target genes acting on different links in the Wnt/β-catenin pathway. Some target genes are integral members of the Wnt/β-catenin pathway, such as FZD4/6, LPR6 and β-catenin, which are up-regulated by lncRNA FYVE, RhoGEF, and PH domain containing 5 antisense RNA 1 (FGD5-AS1) 30 and lncRNA distal-less homeobox 6 antisense RNA 1 (DLX6-AS1) 31 to enhance Wnt signaling. Other target genes indirectly affect the Wnt/β-catenin pathway. Cyclin dependent kinase 14 (CDK14, PFTK1), a serine/threonine protein kinase that can induce phosphorylation of LRP5/6 to activate the Wnt/β-catenin pathway 32, 33, can be up-regulated by lncRNA H19 imprinted maternally expressed transcript (H19) 34. In the nucleus, forkhead box M1 (FOXM1), forkhead box O1 (FOXO3), pygopus family PHD finger 2 (PYGO2) and DNA topoisomerase II alpha (TOP2A) can regulate the transcription of Wnt/β-catenin pathway downstream genes through interacting with β-catenin. Inhibiting the original activity of lncRNAs by binding specific proteins is another way to regulate the Wnt/β-catenin pathway. Both lncRNA long intergenic non-protein coding RNA 01614 (LINC01614) 35 binding GSK‑3β and lncRNA LINC01197^36^ binding β-catenin can affect the original function of the protein to regulate Wnt signal transduction.

What is special in these studies is the regulation of Wnt/β-catenin pathway by lncRNAs through enhancer of zeste 2 polycomb repressive complex 2 subunit (EZH2) and human antigen R (HuR). EZH2 is a catalytic subunit of polycomb repressive complex 2 (PRC2) that can restrain transcription of target genes by triggering trimethylation of methylation of histone H3 at lysine 27 (H3K27me) 37, 38. LncRNA LINC01133 can recruite methylated EZH2 to mediate histone methylation and up-regulate dickkopf Wnt signaling pathway inhibitor 1 (DKK1) 39 and AXIN2^40^ promoter methylation, which inhibited DKK1 and AXIN2 to activate Wnt/β-catenin signaling pathway. The homeobox transcript antisense intergenic RNA (HOTAIR) is one of the most extensively studied lncRNAs found dysregulated in human cancer. Although the mechanism of lncRNA HOTAIR in PC is unknown, HOTAIR can increase the radioresistance of PC cells by down-regulating Wnt inhibitory factor 1 (WIF1) 41, which inhibits the activation of Wnt/β-catenin signaling pathway by binding Wnt protein and inhibiting its signal transduction activity in the intercellular space 42. In esophageal squamous cell carcinoma cells 43 and human chondrosarcoma cells 44, HOTAIR inhibits WIF1 expression by promoting trimethylation of H3K27 in the WIF1 promoter, thereby activating Wnt/ β-catenin pathway. In PC, HOTAIR may also regulate WIF1 in a similar way.

HuR is an RNA binding protein that binds to adenylate/uridylate-rich regions primarily in the 3′ UTR and regulates mRNA stability and translation 45. In PC, the combination of tumor suppressive lncRNA on chromosome 8p12 (TSLNC8), HuR and β-catenin mRNA can promote β-catenin translation and thus activate Wnt signaling 46. Cellular nucleic acid binding protein (CNBP) is a conserved single-stranded nucleic acid-binding protein that acts as both a transcription regulator and a translational regulator 47. Protein tyrosine kinase 7 (PTK7), T-cell-specific transcription factor 4 (TCF4) and CDK14 are direct transcriptional targets of CNBP, which can directly or indirectly participate in Wnt/β-catenin pathway regulation 48. In PC, CNBP could recognize N6-methyladenosine (m6A) lncRNA WT1 associated protein pseudogene 1 (WTAPP1) and recruit HuR to promote WTAPP1 stability. Furthermore, WTAPP1 can enhance WTAP translation by recruiting eukaryotic translation initiation factor 3 subunit B (EIF3B) to WTAP mRNA, induce carcinogenic Wnt signaling and promote PC progression 49. Interestingly, the m6A modification of RNA is dependent on dedicated methyltransferases (METTL), the core of which is the METTL3-METTL14-WTAP complex 50. Then m6A-modified WTAPP1 can promote WTAP expression levels, and WTAP may also form methyltransferase to promote m6A modification of WTAPP1. There may be a positive feedback regulation between WTAPP1 and WTAP. Furthermore, lncRNA family with sequence similarity 83 member H antisense RNA 1 (FAM83H-AS1) is similar to TSLNC8 and FGD5-AS1 in that it can up-regulate the level of β-catenin to activate Wnt/β-catenin signaling, but the difference is that FAM83H-AS1 induces FAM83H expression by stabilizing FAM83H mRNA, thus enhances the ability of FAM83H binding to β-catenin and inhibiting its degradation, and ultimately promotes the proliferation, invasion and metastasis of PC cells 51. The precise mechanism by which FAM83H-AS1 stabilizes FAM83H mRNA is currently unknown, but in ovarian cancer, FAM83H-AS1 could interact with HuR and increase the stability of HuR protein, which has certain guiding significance 52.

### LncRNAs regulate autophagy pathway in PC

There are two sides in the process of transformation and malignant development of cancer cells by autophagy. On the one hand, autophagy removes damaged organelles, peroxides, endogenous bacteria and viruses, prevents normal cells from producing excessive oxidative stress damage to DNA, maintains normal metabolic level, and thus maintains homeostasis of intracellular environment and prevents malignant transformation; on the other hand, while maintaining cell homeostasis, autophagy can also remove various tumor suppressor factors and even therapeutic drugs, increasing drug resistance, which is conducive to the maintenance of tumor and its malignant process^53^-^55^. Current studies have shown that the level of autophagy in PC cells is universally elevated which is highly activated autonomously in the late stage from the formation of intraductal tumors to PC, and a high level of autophagy is required for sustained malignant growth *in vivo* and *in vitro* 56.

The high level of autophagy is associated with the abnormal expression of MIT/TFE family in PC, among which melanocyte inducing transcription factor (MITF), transcription factor binding to IGHM enhancer 3 (TFE3) and transcription factor EB (TFEB) are strongly correlated with autophagic lysosomal characteristics 57, 58. Under nutrient replete conditions, mechanistic target of rapamycin complex 1 (mTORC1) at the lysosomal membrane phosphorylates MIT/TFE proteins, leading to their association with 14-3-3 proteins and retention in the cytoplasm, whereas mTORC1 inactivation upon starvation allows its nuclear translocation 59-62. Autophagy related (ATG) genes regulated by different MIT/TFE family members varied, but the genes ATG9B, ATG16L1, GABA type A receptor associated protein like 1 (GABARAPL1), WD repeat domain, phosphoinositide interacting 1 (WIPI1), and UV radiation resistance associated (UVRAG), which can enhance the biosynthesis of autophagosomes and lysosomes and activate their function, were more than 2-fold expressed when MITF, TFE3, or TFEB were overexpressed, indicating the vital role of the MIT/TFE family in autophagy induction^62^. However, in PC, the nucleoplasmic transport protein, importin 8 (IPO8) can bind to stabilize MIT/TFE factors and translocate to the nucleus, leading to activation of transcription of target genes 57. Current studies on lncRNAs regulating autophagy pathway in PC are shown in the following table (Table 2).

In the research of autophagy regulation in PC, most lncRNAs also play a role by sponging miRNA to stabilize mRNA. For lncRNAs that directly regulate autophagy pathway factors, similar to the regulation of Wnt pathway, lncRNA plasmacytoma variant translocation 1 (PVT1) also regulates autophagy through the target miR-619-5p 20. ATG14 could be down-regulated by miR-619-5p but also activate autophagy by binding to PVT1. Vacuole membrane protein 1 (VMP1) is the downstream target of hypoxia inducible factor 1 subunit alpha (HIF-1α), which can promote the separation of the isolation membrane from the endoplasmic reticulum, and then form free autophagosomes 63. On the other hand, PVT1 can promote autophagy and reduce gemcitabine sensitivity in PC by regulating the miR-143/HIF-1α/VMP1 axis 64. In addition to ATG14, there are lncRNAs that affect autophagy through ATG7. HOTAIR can promote autophagy and down-regulate the radiosensitivity of PC cells by targeting ATG7. In liver ischemia-reperfusion injury 94 and acute lung injury 95, HOTAIR can up-regulate ATG7 and promote autophagy by sponging miR-17-5p and miR-20b-5p. As a key component of the tumor microenvironment (TME), cancer-associated fibroblasts (CAFs) have complex functions to protect cancer cells, which are generally believed to promote cancer and may also inhibit tumor progression in some circumstances 65, 66. In PC cells, lnc-FSD2-31:1 can down-regulate miR‐4736 in extracellular vesicles and up-regulate ATG7, the target of miR‐4736 in CAF, leading to promotion of CAF autophagy and inhibition of CAF fibrosis 67.

Among the factors that indirectly regulate the autophagy pathway, the regulation of HMGB1(high mobility group box 1) and TWIST1(twist family bHLH transcription factor 1) on autophagy is controversial. In the study of Chen *et al.* 68, the knockdown of lncRNA cyclin dependent kinase inhibitor 2B antisense RNA 1 (ANRIL) up-regulated miR-181a and down-regulated HMGB1 at mRNA and protein levels, which made cancer cells sensitive to gemcitabine and showed inhibition of tumor activity and promotion of autophagy. Interference of miR-181a and overexpression of HMGB1 showed opposite biological effects. Therefore, HGMB1 appears to promote PC progression and inhibit autophagy. As a cancer-promoting factor, HMGB1 is also regulated by lncRNA small nucleolar RNA host gene 16 (SNHG16)/miR-218-5p 69 and lncRNA zinc finger E-box binding homeobox 2 antisense RNA 1 (ZEB2-AS1)/miR-204 70 axis in PC. Extracellular HMGB1 can regulate inflammation and PC progression through the receptor of advanced glycosylation end-product specific receptor (AGER) 71, 72 and Toll-like receptor 4 (TLR4) 73. However, in the nucleus, HMGB1 can repair damaged DNA 74. At the same time, low expression of HMGB1 can promote the progression of PC, and the decreased expression of HMGB1 in the pancreas is related to poor survival 75. The reason may be associated with the subcellular localization of HMGB1. Under normal conditions, most HMGB1 is localized in the nucleus, and there is little HMGB1 in the cytoplasm. While under various stresses, HMGB1 is transferred from the nucleus to the cytoplasm, and its extracellular transport mainly depends on the active secretion of living inflammatory cells (such as macrophages) or the passive release of necrotic cells 76. In the aspect of autophagy, HMGB1 is generally regarded as an autophagy inducing factor 77, and cytosolic HMGB1 promotes autophagy by directly binding to beclin-1^78^, contrary to the phenomenon studied by Chen *et al.* 68. This may be related to the potential role of lncRNAs.

TWIST1 is a transcriptional regulator that has been identified to play vital roles in angiogenesis, chemotherapy resistance, metastasis, senescence and stemness in various cancers, including PC 79, 80. The relationship between TWIST1 and autophagy is peculiar. Autophagy deficiency can up-regulate TWIST1^81^, P62 (Sequestosome 1, SQSTM1) can bind and stabilize TWIST1 82, and the formation of B-cell lymphoma 2 (BCL2)/TWIST1 complex can promote the nuclear transport of TWIST1 83. In contrast, TWIST1 silencing can activate AMP-activated protein kinase (AMPK) and inhibit mTOR signaling 84, and its target gene eva-1 homolog A (EVA1A) can promote endothelial cell apoptosis and inflammatory activation through autophagy regulation 85. LncRNA leucine zipper tumor suppressor 1 antisense RNA 1 (LZTS1-AS1) promotes the proliferation, metastasis and oncogenicity of PC cells and inhibits autophagy through LZTS1-AS1/miR-532/TWIST1 axis 86. The mechanism of TWIST regulating autophagy needs further study.

In addition to macroautophagy, mitophagy in PC cells can also be regulated by lncRNAs. The MAPK/ERK pathway is closely related to mitochondrial dynamics 87, which in turn is related to tumor progression 88. In PC, lncRNA urothelial cancer associated 1 (UCA1), which is up-regulated and can enhance the migration ability of cancer cells, regulates mitochondrial dynamics through the activation of MAPK/ERK pathway, including up-regulating mitochondrial membrane potential, enhancing mitochondrial fusion and reducing mitochondrial fission to inhibit mitophagy 89.

LncRNAs can also regulate autophagy through HuR in ways that do not regulate autophagy through miRNAs. TIA1 cytotoxic granule associated RNA binding protein (TIA1) is the same RNA binding protein (RBP) as HuR and has similar regulatory effects on RNA 90, 91. Li *et al.* 92 illustrated that knockdown of lncRNA metastasis associated lung adenocarcinoma transcript 1 (MALAT1) reduced HuR expression, and a direct interaction between MALAT1 and HuR was found. On the other hand, knockdown of MALAT1 had no significant effect on TIA1 accumulation, but enhanced its activity after transcription, while suppressing the expression of autophagy. Therefore HuR can regulate autophagy as an endogenous messenger between MALAT1 and TIA1 to influence tumor proliferation and metastasis. Unfortunately, this article did not validate the interaction between HuR and TIA1, but other studies have shown that HuR can contribute to the maintenance of elevated TIA1 mRNA levels and therefore maintain TIA1 expression by binding to TIA1 3' UTR 93.

**Figure 2:**
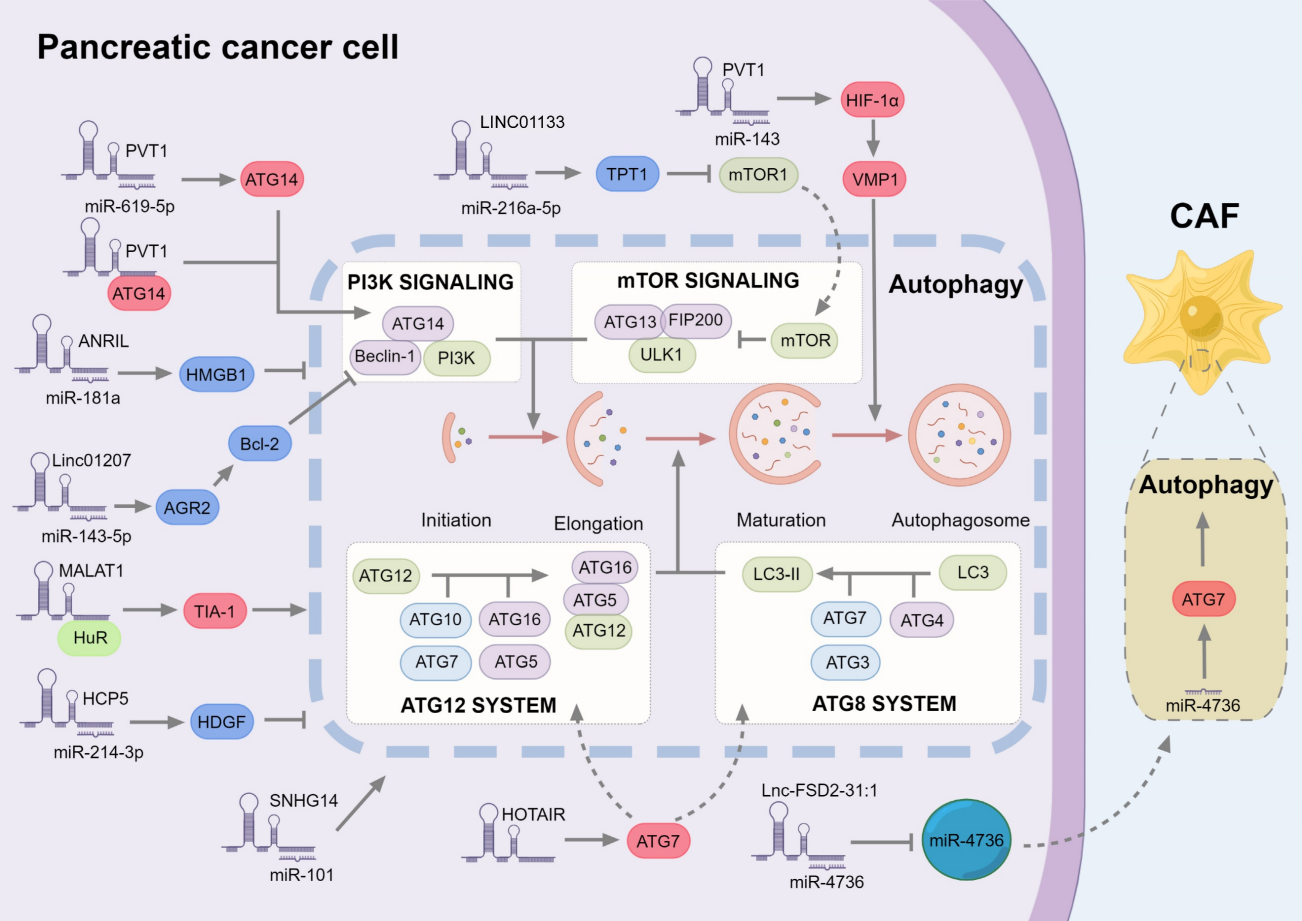
** The role of lncRNAs in regulating autophagy in pancreatic cancer.** The autophagic process is divided into multiple systems that regulate autophagosome formation at different stages. Most lncRNAs also regulate autophagy by sponge miRNA, in which the factors promoting autophagy are marked in red, and the inhibitors are marked in red. Among them, PVT1 can not only promote the expression of ATG14, but also directly bind to ATG14 to promote the formation of autophagosomes. In addition, PVT1 further increased the expression of VMP1 by up-regulating HIF-1α, and VMP1 promoted the separation of the isolation membrane from the endoplasmic reticulum, thereby forming free autophagosomes. Finally, although Lnc-FSD2-31:1 inhibits miR-4736, miR-4736 regulates autophagy of CAFs in the form of exosomes.

In general, although most lncRNAs regulate autophagy in different directions, they generally promote the process of PC, which is related to the duality of autophagy, and the generation of drug resistance is often related to high levels of autophagy. LncRNA can regulate autophagy in a similar way to Wnt/β-catenin pathway, mainly as ceRNA binding miRNA to regulate downstream targets (Fig. 2).

### LncRNA acts as a cross node in autophagy and Wnt/β-catenin pathways

#### Molecular basis between Wnt/β-catenin pathway and autophagy

Wnt/β-catenin pathway and autophagy are important pathways to regulate cell physiological processes and maintain cell homeostasis. In addition to the PI3K-AKT-mTOR pathway, AMPK pathway and EGFR pathway, which can simultaneously regulate Wnt/β-catenin pathway and autophagy 94-97, there is signal crosstalk between the two pathways, and their components also interact with each other at the molecular level to affect their activity and protein level.

β-catenin is a transcriptional regulator downstream of the Wnt/β-catenin pathway, which negatively regulates autophagy and down-regulates the expression of P62 98-100. Under starvation, microtubule associated protein 1 light chain 3 (LC3) directly can target β-catenin for autophagic degradation, resulting in reduced binding of β-catenin to the P62 promoter and increased transcription of P62 to promote autophagy 98, 101. In addition, BCL2, an important target gene of β-catenin 97-99, inhibits beclin-1-dependent autophagy 102. Inhibition of Wnt/β-catenin signaling pathway in PC can down-regulate BCL2, destroy mitochondrial homeostasis, and inhibit tumor growth and development 103.

In the Wnt/β-catenin pathway, GSK‑3β is recruited by AXIN to form a complex with APC and CK1α to phosphorylate β-catenin, leading to its ubiquitination and degradation by the proteasome 104, 105. In the autophagy pathway, GSK‑3β phosphorylates unc-51 like autophagy activating kinase 1 (ULK1) or acetylates ULK1 through the GSK‑3β-TIP60 (lysine acetyltransferase 5, KAT5)-ULK1 axis to activate autophagy 106-108. Meanwhile, AMPK and GSK3 coordinate to phosphorylate tuberous sclerosis complex (TSC) to inhibit mTOR signaling and promote lysosomal acidification 109-112. In addition, GSK3 regulates FOXK1 phosphorylation and inhibits nuclear translocation of FOXK1^113^, ^114^, while FOXK1 inhibits the expression of autophagy genes through recruitment of Sin3A complex 115, 116.

Dishevelled segment polarity protein (DVL) is involved in both canonical and noncanonical Wnt/β-catenin signaling pathways and DVL is recruited to the plasma membrane to bind FZD receptors to initiate Wnt/β-catenin signaling 117, 118. In the autophagy degradation pathway, DVL can directly bind to LC3 and be degraded by P62, thereby inhibiting Wnt signaling 119. The function of DVL is also related to ULK1 kinase activity. DVL can be phosphorylated by ULK1, and Wnt5a-mediated autophagy promotes bacterial clearance in macrophages dependent on DVL and ULK1 120, 121. DVL can also enter the nucleus to form protein complexes with β-catenin and TCF, and induce the transcriptional expression of target genes 122, while DVL phosphorylation by ULK1 inhibits the formation of DVL complexes 121.

In general, the Wnt/ β-catenin pathway and autophagy are mutually inhibitory in normal cells. But the Wnt/β-catenin pathway and autophagy are highly activated in PC compared with normal tissues, and the proliferation, migration, invasion, EMT (epithelial-mesenchymal transition) and drug resistance of cancer cells all depend on high levels of Wnt and autophagy 25, 123. There are other factors affecting both Wnt/β-catenin pathway and autophagy in PC. A tandem mechanism between Wnt/ β-catenin signaling and autophagy has been reported to regulate the progression of PC 20, 95, 124, 125. According to our previous experimental results and research analysis, lncRNA has the potential to simultaneously regulate Wnt/β-catenin pathway and autophagy.

#### Competitive endogenous RNA hypothesis

The ceRNA hypothesis considers miRNAs as miRNA recognition elements (MREs) that bind to RNA transcripts via complementary sequences 126. All types of RNA transcripts have different MRE binding sites, and they can communicate with each other according to the shared MRE, and interact with each other to affect the properties and functions of RNA transcripts 126.

In addition to miR-619-5p and miR-143 mentioned above, PVT1 can also regulate the progression of PC through PVT1/miR-20b/CCND1 (cyclin D1) 127 and PVT1/miR-519d-3p/HIF-1α 128 axes. This means that the same lncRNA can regulate the same mRNA through different MREs, and also can regulate different mRNAs through the same MRE. MiR-195-5p as a communication factor, BRAF-activated non-protein coding RNA (BANCR) can activate the Wnt/β-catenin sighting pathway through miR-195-5p 129. Similarly, miR-195-5p is the target of lncRNA LINC00473, which can drive the progression of PC 130, and WIPI2, a key autophagy regulator 131, can be up-regulated by lncRNA ceramide synthase 6 antisense RNA 1 (CERS6-AS1) to regulate the migration and apoptosis of PC cells via miR-195-5p 132. In summary, a huge communication network is formed between lncRNA-miRNA-mRNA.

Although the ceRNA hypothesis has several limitations: miRNAs target a variety of RNAs, they exert varying degrees of repression on all of them. The differences in RNA types, expression levels, and subcellular localization among different types of cells make ceRNA networks complex and diverse 126, 133. These do not negate the reference value of ceRNA hypothesis. Key lncRNAs and miRNAs can still be found in the ceRNA network of PC cells.

#### Protein-lncRNA interaction

Protein-RNA interaction is universal 134-136. In addition to acting as sponges for miRNAs, lncRNAs can also bind individual proteins or protein complexes and regulate their function 135. Among them, lncRNAs can directly bind to proteins to induce them to target specific sites, and can also serve as scaffolds for the assembly of protein complexes 136. We summarized three types of proteins, which can be regulated by lncRNA, have the potential to simultaneously regulate autophagy and Wnt/β-catenin signaling pathways, and are related to the formation of drug resistance in PC.

#### HuR

RBP can control the metabolism of massive transcripts and is a key factor that regulates gene transcription to translation 137. HuR is one of the most prominent and well-studied factors in RBPs 138, 139. HuR is mainly localized in the nucleus, while the abundance in the cytoplasm varies with the cell cycle, starting to increase at S phase until the cell enters the G2/M phase and decreasing again when the cell re-enters the G1 phase 140. The nucleo-cytoplasmic shuttling ability of HuR is closely related to its physiological function 138.

In PC, HuR would target specific mRNAs in response to external stresses such as gemcitabine 141, hypoxia 142, apoptosis 143, hypoglycemia 144, DNA damage 145, 146, and poly ADP-ribose polymerase (PARP) inhibitors 146. PC cells interfered with HuR siRNA had decreased migratory ability and the tumors formed *in vitro* became smaller 147. The knockdown of HuR induced more PC cell death, while the xenograft tumor experiment using HuR knockout PC cell lines showed no growth at all of the subcutaneous tumors 148. To verify that the absence of HuR resulted in the failure of tumorigenesis, PC cells supplemented with HuR cDNA restored the tumorigenic ability in a nude mouse model 148.

In research on lncRNA, in addition to TSLNC8, MALAT1, and WTAPP1 mentioned earlier, HuR can stabilize lncRNA DNA damage inducible transcript 4 antisense RNA 1 (DDIT4-AS1) by binding to the m6A site and up-regulated DDIT4-AS1 can mediate downregulation of DDIT4 mRNA through upstream frameshift 1 (UPF1), an RNA helicase, thereby activating the mTOR signaling pathway, enhancing PC dryness and inhibiting chemical sensitivity to gemcitabine 149. For mRNA, lncRNA nicotinamide nucleotide transhydrogenase antisense RNA 1 (NNT-AS1) can recognize and stabilize m6A-modified integrin subunit beta 1 (ITGB1) mRNA through METTL3-HuR to activate the MAPK/ERK/PDL1 (programmed cell death 1 ligand 1) signaling pathway and finally promote the immune escape of PC cells 150. In PC, HuR has the potential to recognize and stabilize m6A-modified RNA. In addition, variant subcellular localization of circATG7 regulated ATG7 mRNA level through different pathways 151. In the cytoplasm, circATG7 stabilizes ATG7 mRNA by sponging miR-766-5p, and up-regulates ATG7 mRNA level by recruiting HuR in the nucleus, which eventually leads to the promotion of PC cell proliferation, metastasis and autophagy 151. These all demonstrated that HuR can regulate Wnt/β-catenin signaling and autophagy through multiple pathways and is related to the progression of PC (Fig. ​3). However, it should be noted that there are abundant modification sites on HuR including phosphorylation, methylation, and ubiquitination, which directly affect HuR subcellular localization and RNA binding activity 138. Further studies are needed to investigate post-translational modification of HuR in PC.

#### FOX transcription factor family

FOX (forkhead box) family proteins are evolutionarily conserved DNA-binding proteins 152, 153. Structurally, FOX proteins possess the conserved winged helix forkhead domain, also known as the DNA-binding domain, for attaching DNA 154. The extra-FOX protein-protein interaction domain interacts with other factors to regulate DNA transcription and repair 155.

Here we focused on the FOXO subfamily and FOXM1(Fig. ​4 and 5). In the nucleus, β-catenin can interact with TCF/LEF to induce transcription of target genes 24, but FOXO can competitively bind with β-catenin to inhibit the activity of β-catenin/TCF 156. Both FOXO1 and FOXO3 are down-regulated in PC and inhibit the progression of PC 157-159. Overexpression of LINC00261 can up-regulate FOXO3, the target gene of miR-552-5p, and inhibit the Wnt/β-catenin signaling pathway in PC 157. LINC01197 is a target gene of FOXO1, which inhibits Wnt/β-catenin signaling activity by transcribing LINC01197, allowing it to bind to catenin and disrupt the interaction of catenin with TCF4 in PC cells 36.

For autophagy, sirtuin 1 (SIRT1) is a deacetylase, which can not only directly regulate the deacetylation of autophagy-related factors, such as beclin-1, ATG5, ATG7 and LC3 to induce autophagy 160-162, but also regulate autophagy through the FOXO family members FOXO1 and FOXO3^163^, ^164^. Sirt1-mediated FOXO1 deacetylation could activate its function and nuclear translocation to ultimately promote autophagy, while activation of FOXO1 can enhance the expression of RAS-related GTP-binding protein 7 (Rab7) 163, a small GTPase that mediates late autophagosome-lysosome fusion 165. In addition, acetylated FOXO1 can directly bind ATG7 to induce autophagy 166. In multiple cell lines, including PC cells, it has been confirmed that miR-138-5p specifically targets SIRT1 3' untranslated region and inhibits autophagy by reducing SIRT1^164^, ^167^, ^168^. Furthermore, knockdown of Rab7 or FOXO1 in PC inhibited the SIRT1-mediated increase of autophagic flux, suggesting that SIRT1 regulates autophagy in PC via FOXO1/Rab7 axis 164. However, miR-138-5p can be spongy by lncRNA H19^169^ and HOTAIR 170, which are up-regulated in PC 41, 171. lncRNA may affect autophagy in PC by regulating SIRT1.

FOXO3 could up-regulate the transcription of multiple autophagy genes, such as ULK2, beclin-1, phosphatidylinositol 3-kinase catalytic subunit type 3 (PIK3C3), BCL2 interacting protein 3 (BNIP3), ATG4B, ATG4C, ATG5, ATG7, ATG12, ATG13, ATG14, ATG16L1, LC3, and GABARAPL1 172-174. FOXO3 deacetylated by SIRT1 can drive the transcription of BNIP3 and induce mitochondrial autophagy 175, 176. In the study of skeletal dystrophic cachexia of PC, SIRT1 can indirectly regulate the expression of FOXO1 and FOXO3 by nuclear transcription factor-kappa B (NF-kB) signaling 177. SIRT1 knockout induced NF-kB signaling and enhanced NADPH oxidase 4 (NOX4) transcription in cachexia muscles caused by PC, leading to increased reactive oxygen species (ROS) levels and FOXO expression 177. Metformin differentially can regulate cellular ROS levels through AMPK-FOXO3-MnSOD (manganese superoxide dismutase, SOD2) pathway, especially in PC cells 178. After combined administration with apigenin, ROS levels were further increased and exerted anticancer activity through DNA damaging-induced apoptosis, autophagy, and necrosis 178.

FOXM1 expression is elevated in a wide range of cancer cell lines and cancer types and can be used as a biomarker for cancer diagnosis, treatment, and prognosis 179-181. In PC, LINC00857 acts as a protein scaffold to bind FOXM1 to ovarian tumor family deubiquitinase ubiquitin aldehyde binding 1 (OTUB1), thereby inhibiting FOXM1 degradation through the ubiquitin-proteasome pathway 182. In the Wnt/β-catenin pathway, FOXM1 competes with FOXO and combines with β-catenin to regulate the transcription of downstream factors, thus modulating Wnt signal 183. In terms of autophagy, FOXM1 is shown to up-regulate LC3-II/LC3-I and beclin-1 and promote autophagy in bladder cancer 184, liver cancer 185, prostate cancer 186, and gastric cancer 187, and it is determined in triple-negative breast cancer cells that FOXM1 directly binds to the promoter of LC3 and beclin-1 genes to promote transcription 188.

#### HIF family

In both primary and metastatic tumors, PC has characteristics of high levels of fibrosis 189. The resulting hyperplasia of connective tissue forms a mechanical barrier around tumor cells, restricting the generation of blood vessels. Moreover, PC cells have a high level of metabolism, so the cancer microenvironmen is severely hypoxic, which is another characteristic of PC 190, 191. The mechanism of biological adaptation to hypoxia is mediated by hypoxia-inducible factor (HIF) 192, which is regulated by a dimer composed of α subunits (Hif-1α, HIF-2α and HIF-3α) and β subunits (HIF-1β) 193. Under normal oxygen conditions, HIF-α protein subunit is unstable and rapidly degraded by proteasome. Under hypoxic conditions, HIF-α is stable and translocates to the nucleus to bind to HIF-1β, where it is induced to the regulatory regions of target genes and modulates their transcription 194. HIF-1α and HIF-2α are two major isoforms in mammalian cells. HIF-1α is widely distributed in almost all types of cells and is also regarded as a widely used hypoxia marker 195, while HIF-2α is expressed in certain cell types such as hepatocytes and endothelial cells 196.

PC cells can adapt to the extreme conditions of hypoxia by activating HIF, which in turn transcribe genes related to angiogenesis and glycolysis 197. According to recent studies, HIF-1α has feedback regulation with various lncRNAs in PC, and promotes PC progression and drug resistance (Fig. ​6). LncRNA CF129145. 1 (CF129), a downstream target gene of HIF-1α, was inhibited by HIF-1α under hypoxic conditions, and CF129 reduction inhibited the degradation of tumor protein P53 (P53, TP53) by makorin ring finger protein 1 (MKRN1) ubiquitination. After P53 translocation into the nucleus and transcription of FOXC2, FOXC2 can bind to the HIF-1α promoter to activate its transcription. Thus, during hypoxia, HIF-1α/CF129/P53/FOXC2 forms a feedback loop and promotes PC progression 198.

LncRNA HIF1A-AS1 is an antisense RNA of HIF-1α, and HIF-1α can also activate HIF1A-AS1 transcription 199. In the cytoplasm, HIF1A-AS1 can induce Y box binding protein 1 (YB1) to interact with the serine/threonine kinase AKT, leading to phosphorylation of YB1 (pYB1). Meanwhile, pYB1 is recruited by HIF1A-AS1 to bind to HIF-1α mRNA, thereby promoting the translation of HIF-1α. Thus, the positive feedback between HIF1A-AS1 and HIF-1α makes them highly expressed in PC and promotes the resistance to gemcitabine 199.

Similarly, lncRNA NR2F1-AS1 is an antisense RNA of nuclear receptor subfamily 2, group F, member 1 (NR2F1), which is the target of NR2F1-AS1 and is positively regulated by NR2F1-AS1. NR2F1 can activate AKT/mTOR pathway and up-regulate HIF-1α in PC. NR2F1-AS1 is also a hypoxia-responsive lncRNA in PC cells, which can be transcription by HIF-1α under hypoxic conditions. Therefore, NR2F1-AS1 forms a positive feedback with HIF-1α via the NR2F1/AKT/mTOR axis 200.

ZEB1(zinc finger E-box binding homeobox 1) is an EMT activator and a key regulator of PC cell plasticity, metastasis and drug resistance 201, 202. By binding with histone deacetylase 1 (HDAC1) and HIF-1α, ZEB1 can inhibit the acetylation of HIF-1α and further maintain the stability of HIF-1α 203. In PC, ZEB1-AS1 is an antisense RNA of ZEB1, which can up-regulate the mRNA and protein levels of ZEB1^203^, and lncRNA ZEB1 transcriptional regulator RNA (BX111, ZEBTR) induces ZEB1 transcription by recruiting YB1^204^. Under hypoxia, both ZEB1-AS1 and BX111 are transcriptized and up-regulated by HIF-1α, which stabilizes HIF-1α through ZEB1, thus forming a positive feedback loop with HIF-1α 203, 204. Moreover, similar to ZEB1, metastasis associated 1 family member 2 (MTA2) can form a complex with HDAC1 to deacetylate and stabilize HIF-1α 205. LncRNA MTA2 transcriptional regulator RNA (MTA2TR) is regulated by HIF-1α transcription in PC and recruits activating transcription factor 3 (ATF3) to the MTA2 promoter to promote MTA2 transcription, forming a HIF-1α/MTA2TR/MTA2 positive feedback loop 206.

In addition to targeting miR-143 64 and miR-519d-3p 128 to regulate HIF-1α expression, there is a positive feedback loop between PVT1 and HIF-1α. 207 On the one hand, PVT1 can bind to the HIF-1α promoter and activate its transcription, and bind to HIF-1α protein to up-regulate the level of HIF-1α after translation. On the other hand, PVT1 is also a downstream target of HIF-1α, and HIF-1α can bind to the PVT1 promoter to activate its transcription. Moreover, the expression level of PVT1 and HIF-1α can stabilize each other after transcription, HIF-1α can down-regulate the attenuation rate of PVT1, and PVT1 can inhibit the proteasome-dependent degradation of HIF-1α. In this way, the positive feedback loop between PVT1 and HIF-1α promotes the progression of PC 207.

HIF-1α has been widely confirmed to promote autophagy 208, 209, and its downstream factor BNIP3 has been identified in a variety of cells 210-213. Furthermore, HIF-1α has been shown to promote the transformation of non-stem cell PC cells into prominin 1 positive (CD133+, PROM1 positive) PC stem cell-like cells under intermittent hypoxia by inducing autophagy 214, and to promote the EMT and metastatic capacity of stem cells 215. On the other hand, HIF-1α is also generally believed to be activated by the Wnt/β-catenin pathway 216, 217 (Fig. ​7). In PC, as a transcriptional chaperone of β-catenin, TCF4 positively can regulate aerobic glycolysis by inhibiting egl-9 family hypoxia-inducible factor 2 (EGLN2), leading to up-regulation of HIF-1α 218. In esophageal squamous cell carcinoma, HIF-1α can directly bind to the promoter region of TCF4 and promote the expression of TCF4^219^. HIF-1α can also directly bind to β-catenin 219-221, enhance the transcriptional activity of HIF-1α 220, 221, but inhibit the interaction between TCF4 and β-catenin, making β-catenin lose the ability to transduce signals 220, 221. In addition to affecting the transcriptional activity of β-catenin, mutant Kirsten rat sarcoma viral oncogene homolog (K-RAS) can up-regulate HIF-1α in PC cells, and HIF-1α overexpression increases the protein level of CDK8. Furthermore, CDK8 stabilizes β-catenin and activates Wnt/β-catenin pathway by regulating AXIN2 and GSK‑3β 222. Similarly, phosphoglycerate mutase 1 (PGAM1), a key glycolytic protein, is significantly overexpressed in PC metastases and associated with poor prognosis 223, while the use of an allosteric PGAM1 inhibitor restrains PC progression 224. Among them, PGAM1 mainly exists in the cytoplasm and cell membrane and interacts with HIF-1α to positively regulate each other. The up-regulated PGAM1 promotes EMT by activating Wnt/β-catenin signaling pathway 223. Thus, there may be complex feedback regulation between HIF and Wnt/β-catenin signaling pathways.

Regarding HIF-2α, CAF cells with specific deletion of HIF-2α inhibited PC tumor progression and growth and increased the survival of experimental mice by 50% 225. Down-regulation of HIF-2α in CAF induced tumor fibrosis and significantly reduced the intratumoral recruitment of immunosuppressive M2 macrophages and regulatory T cells, and improved the immunosuppressive effect of TME 225. The interaction between HIF-2α and β-catenin in PC leads to increased activity of classical Wnt/β-catenin, and also promotes HIF-2α transcriptional activity by stabilizing HIF-2α 226. HIF-2α is associated with the early development of PC. In normal human pancreas, HIF-2α is easily degraded to a very low level, but hypoxic conditions induce the stabilization of HIF-2α, leading to the development of chronic pancreatitis 227. In the context of oncogenic K-RAS, pancreatic cells further develop into cysts similar to mucinous cystic neoplasms 227, the formation of which is associated with the activation of Wnt/β-catenin signaling 228. Knockdown of HIF-2α in low-grade pancreatic intraepithelial neoplasia (PanIN) increased the number of cell lesions, but these lesions failed to progress to high-grade PanIN and showed decreased protein levels of β-catenin and drosophila mothers against decapentaplegic protein 4 (SMAD4) 229. Interestingly, the expression of β-catenin was negatively regulated by SMAD4 230, and HIF-2α could regulate the expression of β-catenin and SMAD4 independently in different ways 229. The two pathways are competitive, and HIF-2α is more likely to up-regulate β-catenin to activate the canonical Wnt/β-catenin signaling pathway in the early progression of PC 229 (Fig. ​7).

### Resistance to gemcitabine

#### Mechanisms of action of gemcitabine

Gemcitabine is the first-line drug for the treatment of advanced PC, and the current research on drug resistance focuses on gemcitabine 12. Gemcitabine is a cytosine nucleoside derivative, also known as 2',2'-difluoro-2'deoxycytidine (dFdC), and its mechanism of action is related to the multiple effects of its intracellular metabolites on DNA synthesis 231. After entering the cell via nucleoside transporters, gemcitabine is progressively phosphorylated to gemcitabine monophosphate (dFdCMP), gemcitabine diphosphate (dFdCDP) and gemcitabine triphosphate (dFdCTP) 231. Among them, dFdCTP can be involved in DNA synthesis. After dFdCTP is incorporated into the DNA chain and ligated with another deoxynucleotide, the DNA strand stops extending, which is called "masked chain termination". 231, 232 Similarly, this effect contributes to the inability of DNA repair enzymes to recognize dFdCTP, which interferes with the normal DNA repair function of cells, so that gemcitabine continues to exert the function of inhibiting DNA synthesis 232. In addition, there are other mechanisms by which gemcitabine interferes with cellular regulation. Different metabolites increase each other's physiological activities and enhance the ability to inhibit cell growth as a whole. This interaction is called "self-enhancement"^232^. dFdCTP competes with deoxycytidine triphosphate (dCTP) for binding to DNA polymerase to inhibit its activity 231. As a ribonucleoside reductase (RR) inhibitor, dFdCDP can regulate RR activity by limiting the formation of nucleoside triphosphate (NTP), which reduces cytidine diphosphate (CDP) to deoxycytidine diphosphate (dCDP)^233^, leading to depletion of the deoxyribonucleotide pool required for DNA synthesis and enhancing the effect of dFdCTP^231^. dFdCTP can also inhibit the effect of deoxycytidine monophosphate deaminase (dCMP) on dFdCMP, preventing its conversion to 2',2'-difluorodeoxyuridine monophosphate (dFdUMP), which is then discharged from cells 231, 234, 235.

#### Regulatory pathways for gemcitabine

In PC, the intracellular metabolism of gemcitabine requires the co-regulation of multiple enzymes, which are regulated by a variety of miRNAs 236. According to the ceRNA hypothesis 126, these miRNAs can be used as the mediators of lncRNA regulation of downstream factors, and lncRNA also has the potential to regulate the metabolism of gemcitabine cells.

Besides these pathways, the susceptibility of PC cells to gemcitabine is inversely proportional to the levels of Wnt/β-catenin and autophagy pathways. LncRNA can affect the resistance of PC cells to gemcitabine through autophagy and Wnt/β-catenin pathways. In addition to PVT1 mentioned above, lncRNA histocompatibility leukocyte antigen complex P5 (HCP5) can act as ceRNA to inhibit the expression of miR-214-3p and target heparin binding growth factor (HDGF) to regulate the proliferation, invasion, migration, apoptosis and autophagy of PC cells, thus promoting gemcitabine resistance 237. In pancreatic stellate cell (PSC), HIF-1α can induce the expression of HDGF and the increased level of HDGF shows anti-apoptotic and pro-fibrotic effects, which can maintain tumor lesions 238. Interestingly, HDGF was more commonly found to regulate Wnt/β-catenin pathway than autophagy in cancer cells 239-242. The expression of Wnt/β-catenin pathway genes was increased in the xenograft model of primary non-small cell lung cancer (NSCLC), while Wnt/β-catenin pathway genes were significantly down-regulated after anti-HDGF treatment, especially Wnt1 and FZD were severely inhibited. HDGF may be a target for inhibiting the proliferation of cancer stem cells and preventing the recurrence of lung cancer after chemotherapy 243.

#### HuR

**Figure 3:**
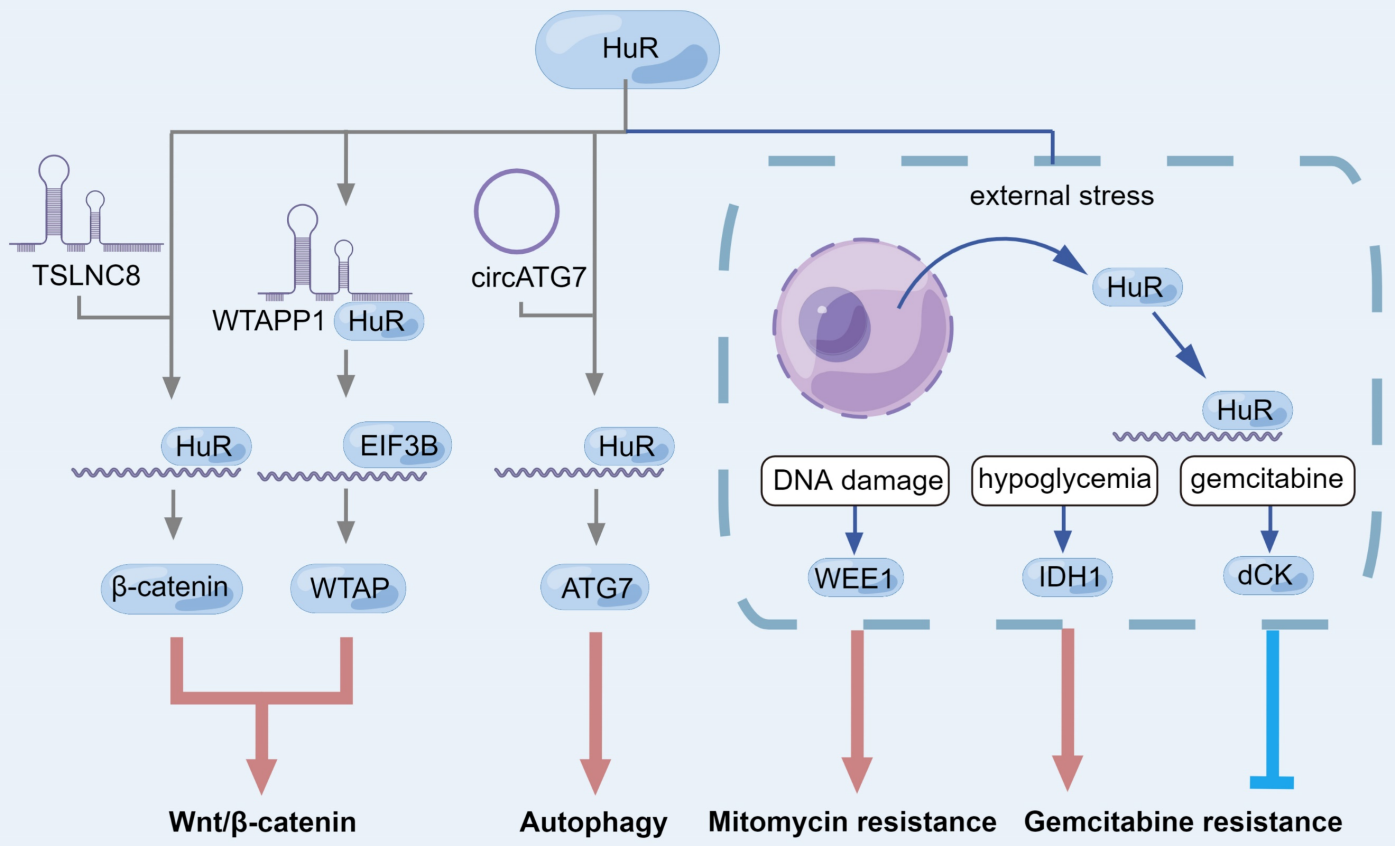
** The role of HuR during Wnt/β-catenin signaling pathway, autophagy and drug resistance in pancreatic cancer.** LncRNA can induce HuR to bind to mRNA to promote the translation of downstream factors, or directly bind to HuR to recruit EIF3B to the mRNA translation initiation site, which ultimately manifests as autophagy and Wnt/β-catenin pathway activation. In response to different external stresses, HuR is translocated from the nucleus to the cytoplasm, where HuR can up-regulate specific factors to regulate drug resistance of pancreatic cancer cells under the conditions of DNA damage, hypoglycemia and gemcitabine treatment.

In addition to being targeted by lncRNAs to regulate autophagy and Wnt/β-catenin in PC, HuR also shows a strong correlation with gemcitabine resistance, which can be used as a marker for the treatment and prognosis of PC 244-246 (Fig. 3).

In PC cells, HuR associates with deoxycytidine kinase (dCK) mRNA, which encodes a metabolic enzyme that can activate gemcitabine 141, 247. Inducible factors including gemcitabine can increase the translocation of HuR from the nucleus to the cytoplasm, leading to the strengthening of the association between HuR and dCK mRNA, resulting in the attenuation of gemcitabine resistance 141, 145, 247, 248. On the other hand, HuR can up-regulate tumor resistance to other chemotherapeutic agents. WEE1 G2 checkpoint kinase (WEE1) mRNA, a mitotic inhibitor kinase, can be stabilized by HuR 145 to regulate DNA damage repair pathways 249, 250. Similarly, when PC cells were under external environmental stress, such as DNA damage, HuR translocalized from the nucleus to the cytoplasm and then up-regulating WEE1. Positive regulation of WEE1 by HuR can increase H2A. X variant histone phosphorylation at serine-139 (γH2AX) levels, induce CDK1 phosphorylation, and promote cell cycle arrest at G2-M transition 145. Different from the effect on gemcitabine, the increase of HuR contributes to DNA damage repair and resistance to cytotoxic therapy 145.

HuR can also affect the drug resistance in PC by regulating glucose metabolism. Although low nutrient levels slow down the growth of PC cells, they promote chemotherapy resistance 144. Acute glucose deprivation can act as a potent stimulus for HuR translocation from the nucleus to the cytoplasm, where HuR stabilizes its target mRNA. These transcripts encode enzymes essential for glucose metabolism, and these targets are essential for the survival of cancer cells in the metabolically impaired TME 251. Silencing HuR attenuated drug resistance in PC cells, and drug resistance would be further reduced under conditions of nutrient deficiency. This is mainly due to the fact that HuR can export from the nucleus to the cytoplasm to stabilize isocitrate dehydrogenase (NADP(+)) 1 (IDH1) expression and enhance ROS scavenging, which enhances the reducing capacity of PC and protects PC under nutrient deficiency 251. Similarly, IDH1 overexpression also enhanced gemcitabine resistance in PC cells 144.

#### FOXO3-FOXM1 axis

FOXO3 and FOXM1 are a pair of transcription factors with opposite functions, which not only compete for binding to promoters of the same DNA motif, but also have opposite transcriptional effects on target genes 252. In addition, FOXM1 is also a downstream target of FOXO3, and FOXM1 is negatively regulated by FOXO3^253^, ^254^. FOXO3-FOXM1 axis is a key regulatory target of cancer drug resistance 255. The dysregulation of FOXO3-FOXM1 axis outside the autophagy and Wnt/β-catenin pathways can lead to drug resistance by regulating drug efflux and DNA repair 255, 256 (Fig. ​8). Membrane ATP binding cassette (ABC) transporters are tightly linked to drug transport, acting as protein pumps for drug efflux that drive the development of multidrug resistance (MDR) in cancer cells 257. FOX proteins are transcription factors that can directly regulate the expression of different ABC proteins, and the target genes of FOXM1 include ABCC4 258, ABCC5 259, 260, ABCC10 261, ABCG2 262, FOXO3 include ABCA6 263, ABCB1 264, FOXO1 include ABCA1 265, ABCA6 263, ABCA9 266, ABCC2 265. Meanwhile, the up-regulated FOX proteins in cancer cells can generate drug resistance in an ABC protein-dependent manner 256, 258-262, 264, 265. On the other hand, chemotherapeutic agents can mediate their cytotoxic and cytostatic functions through FOXO3 and FOXM1. For example, paclitaxel induced FOXO3 nuclear translocation to mediate its cytotoxicity and in turn promoted breast cancer cell death due to the fact that paclitaxel can promote the nuclear translocation of FOXO3 by activating c-Jun NH2 terminal kinase 1/2 (JNK1/2) in combination with inhibiting the AKT pathway and activating the pro apoptotic molecule BCL2-interacting mediator of cell death (BIM, BCL2L11) to trigger apoptosis 267, 268. Similarly, paclitaxel can down-regulate the mRNA and protein levels of FOXM1 and induce mitotic arrest and senescence in cancer cells partly by down-regulating FOXM1 269.

In PC, gemcitabine resistance is associated with FOXM1 stability 270, 271. Ubiquitin C-terminal hydrolase L3 (UCHL3) as a deubiquitinating enzyme is able to inhibit FOXM1 ubiquitination and degradation 270, while Human leukocyte antigen F-associated transcript 10 (FAT10) as a ubiquitin like protein is able to inhibit FOXM1 ubiquitination and stabilize FOXM1 expression by competing with ubiquitin for binding to FOXM1 271. The down-regulation of UCHL3 and FAT10 increased FOXM1 ubiquitination, and also promoted the sensitivity of PC cells to gemcitabine 270, 271. FOXM1 expression was also up-regulated in the gemcitabine-resistant cell line model 272.

For other chemotherapeutic drugs, such as paclitaxel, FOXM1 can induce paclitaxel resistance in PC through different pathways. Prohibitin 1 (PHB1) is a downstream factor of FOXM1, which can recruit Raf-1 proto-oncogene (RAF1) to Caveolin-1-enriched lipid rafts and activates the RAS-RAF-MEK-ERK pathway leading to drug resistance 273. In the cytoplasm, FOXM1 can bind to PHB1 and stabilize PHB1 to shift it to the cell membrane. PHB1 depletion can also down-regulate the level of FOXM1, and there is a positive feedback regulation of FOXM1/PHB1/RAF-MEK-ERK 273, 274. In addition, ABCA2 is also regulated by FOXM1/PHB1/RAF-MEK-ERK pathway. Overexpression of FOXM1 or depletion of PHB1 affected ABCA2 protein and mRNA levels 273. The expression levels of FOXM1, PHB1, ABCA2 and p-ERK1/2 were up-regulated in PC cells and paclitaxel-resistant cell lines, and were also proportional to the drug dose 273. Genistein is a natural isoflavone found in legumes with antitumor effects 275. It has been reported to affect the incidence of PC 276, 277 and inhibit the growth of cancer cells *in vitro* and *in vivo* 278, which is a potential chemopreventive and therapeutic agent for PC. After treatment with genistein, the expression of FOXM1 and its downstream genes was down-regulated, and the growth and invasion of PC cells were inhibited 279. Whereas overexpression of FOXM1 reduced genistein-induced cell growth inhibition and apoptosis 279. NOSH-aspirin, a novel anti-inflammatory agent, also has anticancer effects and can significantly reduce the growth and progression of PC 280, 281. The high level FOXM1 of tumors in a xenograft mouse model of PC was also observably inhibited by NOSH-aspirin 282. Dacomitinib can inhibit the growth and proliferation of PC cells by down-regulating FOXM1 and its downstream targets, such as polo-like kinase 1 (PLK1), survivin, cyclin B1 (CCNB1), c-Myc and aurora kinase B (AURKB) 283.

In contrast to FOXM1, FOXO3 is regarded as a tumor suppressor and the overexpression of FOXO3 can inhibit the proliferation, tumorigenic potential and invasiveness of various cancer cells 284. Similarly, downregulation of FOXO3 can promote PC development 158. But the difference is that the role of FOXO3 on drug sensitivity may be multifaceted.

In cholangiocarcinoma (CCA), LINC01714 was shown to be recurrently down-regulated in clinical samples and significantly correlated with overall survival in patients, and overexpression of LINC01714 inhibited the proliferation, migration and invasion of cancer cells both *in vitro* and *in vivo* 285. Moreover, LINC01714 interacted with FOXO3 with increased FOXO3 protein level but decreased FOXO3 phosphorylation at Ser318 site. Interestingly, LINC01714 enhanced gemcitabine sensitivity of CCA tumor cells by regulating FOXO3 phosphorylation 285. This may be related to the output of FOXO3 phosphorylation from the nucleus and the loss of its transcriptional activity 286. In NSCLC, both tripartite motif containing 22 (TRIM22) and troponin C1 (TNNC1), downstream factors negatively regulated by FOXO3, are up-regulated in cancer cells and gemcitabine resistant cell lines and confer gemcitabine resistance by protective autophagy 287, 288.

In different cancers, the variant expression of FOXO3 has variant effects on prognosis. High expression of FOXO3 has a favorable prognosis in acute myeloid leukemia 289, breast cancer 290, bladder cancer 291, gastric cancer 292, nasopharyngeal carcinoma 293 and human ovarian cancer 294. But in hepatocellular carcinoma 295, 296 and PC 297, high levels of activated FOXO3 lead to poor patient prognosis. This discrepancy may be due to P53 mutations. In the case of P53 mutations, FOXO3 acts as a tumor suppressor, and wild-type P53 alters the site at which FOXO3 recognizes target promoters, thereby inhibiting FOXO3 induced apoptosis and instead potentiating chemoresistance and survival of cells 298.

For PC, FOXO3 is a target of miR-223, and downregulation of FOXO3 by miR-233 leads to the proliferation, apoptosis, and cisplatin resistance in cancer cells 299. Furthermore, FOXO3 is essential for cluster of differentiation-44 (CD44) expression and cancer stem cell (CSC) properties, and inhibition of FOXO3 by cyclic guanosine monophosphate (cGMP) 297 as well as through FOXO3/LKB1/AMPK/PGC-1β (peroxisome proliferator-activated receptor gamma, coactivator 1 beta)/PDHA1 (pyruvate dehydrogenase E1 subunit alpha 1)/CD44 axis influences the CSC phenotype of PC cells 300. FOXO3 to maintain CD44 expression enables CSCs to acquire drug resistance 297, 300. Cardamonin (CAR), a flavonoid present in the genus arangal, inhibits PC cell growth and promotes apoptosis 301, 302. After controlled experiments, CAR can promote the chemosensitivity of PC cells to gemcitabine, and cell viability is further decreased after combined use of gemcitabine. These functions are achieved by CAR through FOXO3 promotion and FOXM1 inhibition 302.

#### Hypoxia signaling pathway

**Figure 9:**
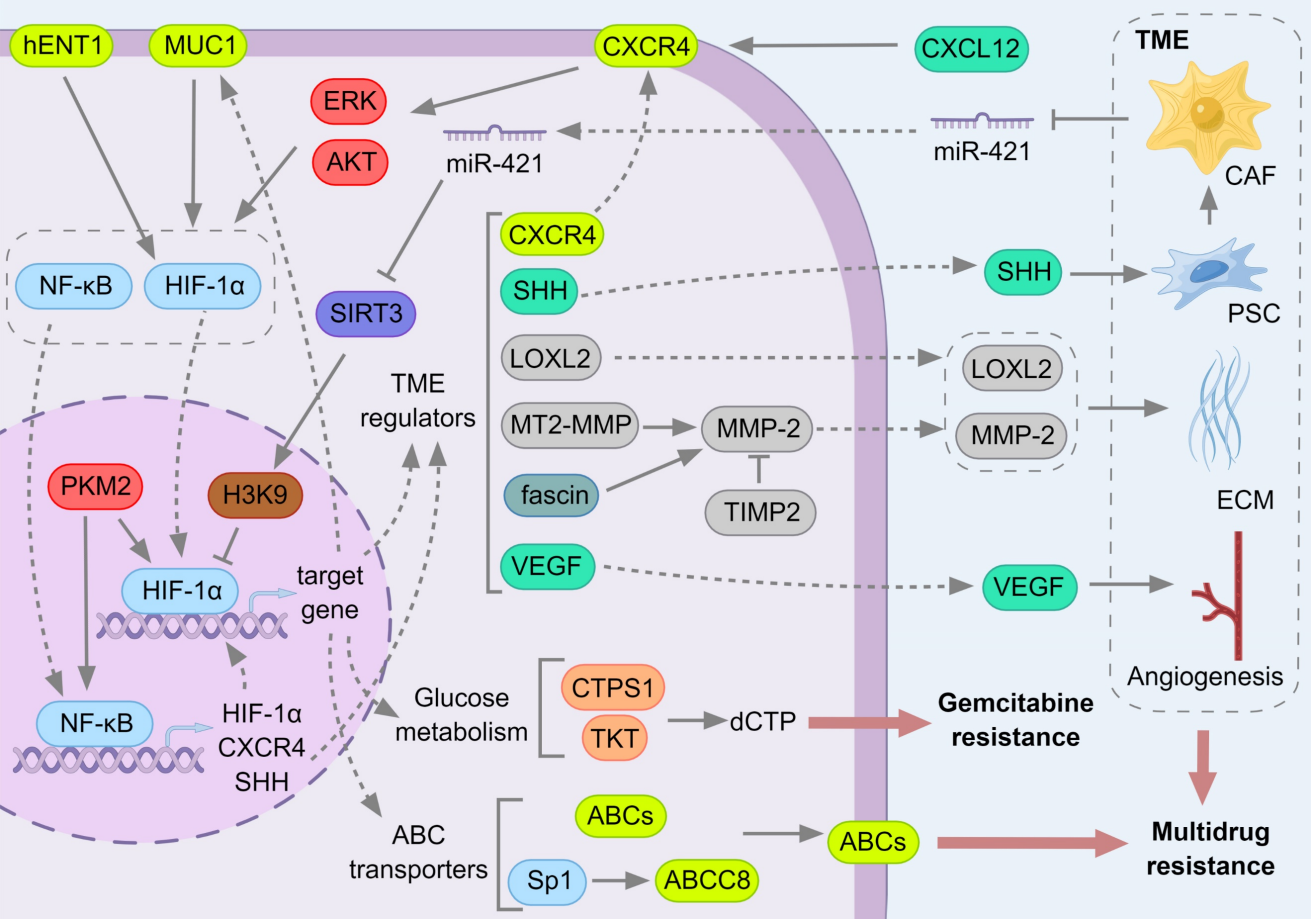
** The role of HIF-1α in regulating drug resistance in pancreatic cancer.** HIF-1α can make pancreatic cancer resistant in three ways. First, multiple ABC transporters are downstream target genes of HIF-1α, and HIF-1α also upregulates the transcription factor Sp1, which transcribes ABCC8. Up-regulation of ABC transporters promote drug efflux and make cells acquire drug resistance. Secondly, HIF-1α can regulate glucose metabolism in pancreatic cancer cells hENT1 promotes the nuclear translocation of HIF-1α, and MUC1 promotes the nuclear displacement of NF-κB and HIF-1α, thereby altering the glucose metabolism level of pancreatic cancer cells. After inducing the transcription of glycolytic genes (TKT and CTPS1), the synthesis of dCTP is increased, which can compete with gemcitabine for DNA synthesis and inhibit the cytotoxicity of gemcitabine. Finally, a variety of proteins in the target genes of HIF-1α are transferred to the cell membrane or secreted to the extracellular to participate in the regulation of tumor microenvironment. Tumor microenvironment affects HIF-1α expression and drug resistance by maintaining hypoxic environment, regulating intercellular signaling and forming physical barriers.

The main role of HIFs in mediating tumor biology is caused by hypoxia 192 and the hypoxic environment of PC is in turn associated with its extreme TME 190, 191. The function of HIFs to induce drug resistance is tightly linked to hypoxia and the TME (Fig. 9). Under hypoxic conditions, HIF-α remains stable and translocates to the nucleus to bind to HIF-1β, which in turn induces downstream target gene transcription 194. Typical among the factors downstream of HIF that have been associated with drug resistance are the ABC transporters, which act as protein pumps to efflux drugs enabling cells to acquire drug resistance 257. Hypoxia-induced HIF-1α and HIF-2α have a promoting effect on the expression of ABC transporters, including ABCB1, ABCB5, ABCC1, and ABCG2, and induce drug resistance in cancer cells 303.

HIF-1α can directly bind to the promoters of ABCC1 304, ABCB1 305, ABCB6 306, ABCA1 307 and ABCG2 308, and HIF-2α can directly bind to the promoter of ABCG2 309 to promote the transcriptional expression of ABC proteins. In addition, HIF-1α can also transcribe specific protein 1 (SP1), which can activate the ABCC8 promoter 310. For PC, HIF-1 has been confirmed to make cancer cells resistant to gemcitabine and 5-Fluorouracil (5-FU) by regulating ABCB1 and ABCG2 308, 311. While quercetin, a flavonoid that can inhibit the efflux activity of ABCB1, in combination with gemcitabine can down-regulate HIF-1α and up-regulate the apoptosis regulator P53, enhancing the cytotoxic activity of gemcitabine 312.

Besides, hypoxia-induced HIF stabilization can reprogram the metabolic way of cancer cells and produce drug resistance. HIF-1 can activate the transcription of glucose transporters and glycolytic enzymes and increase glucose metabolism through the glycolytic pathway, but reduce glucose entry into the tricarboxylic acid cycle (TCA cycle) 313, and this metabolic pattern is beneficial to cancer cell proliferation. Under hypoxic and glucose deprivation conditions, HIF can activate anaerobic metabolism of PC cells and inhibit their apoptosis 314. Meanwhile, mucin 1 (MUC1), a polymorphic mucin-like protein that is overexpressed in PC, can stabilize HIF-1α and promote HIF-1α recruitment to glycolytic gene promoters (transketolase (TKT) and CTP synthase 1 (CTPS1)) in a hypoxia-dependent manner 315. MUC1 is also a target gene of HIF-1α 316, and MUC1 and HIF-1α can regulate each other in a positive feedback manner. MUC1 and HIF-1α can synergistically regulate glucose metabolism and pyrimidine biosynthesis in gemcitabine-resistant PC cells, resulting in increased nucleotide synthesis and accumulation of dCTP, which can cause competitive inhibition of active gemcitabine, thus producing gemcitabine resistance 317.

The level of human equilibrative nucleoside transporter 1 (hENT1) is tightly associated with the sensitivity of PC cells to gemcitabine and can be used as a biological indicator to predict the efficacy and prognosis of gemcitabine 318-321. HENT1 was down-regulated in gemcitabine resistant PC cells, and overexpression of hENT1 significantly reversed chemoresistance. Specifically, hENT1 can induce the development of drug resistance by regulating glucose transport and glycolysis through HIF-1α and c-Myc, low hENT1 and c-Myc expression in drug-resistant cells, highly active HIF-1α potentiates glycolysis and generates chemoresistance, whereas overexpression of hENT1 elevated c-Myc expression, suppressed HIF-1α, restored glucose transport and glycolysis in cells and reversed gemcitabine induced drug resistance 322.

The effects of TME and PC cells are reciprocal. In addition to maintaining the hypoxic environment of cancer cells to stabilize HIF-1α and activate its downstream target genes, CAF also regulate cancer cell metabolism through paracrine pathways. Exosome miR-421 secreted by CAF can down-regulate SIRT3 in PC cells 323. SIRT3 is a target of miR-421 and also located upstream of HIF-1α. It can inhibit the expression and activity of HIF-1α in different cancer cells through AMPK/mTOR axis 324, FOXO3 325 and reducing ROS 326. In PC, SIRT3 inhibited the transcription of HIF-1α by deacetylating acetylation of Histone H3 at lysine9 (H3K9ac), and miR-421 with high expression of CAF promoted the proliferation of PC cells 323.

In contrast, HIF-1α can generate a desmoplastic response that solidifies the TME of PC 327. The sonic hedgehog ligand (SHH) is a member of the hedgehog proteins and a frequently used signaling transmitter in mediating intercellular communication 328. In PC, SHH can regulate the physiological activities of PC cells, including desmoplasia, cancer cell metastasis, and lymphatic vessel formation, through paracrine secretion 329, 330. It has been reported that SHH is expressed in PC cells in a HIF dependent manner and induces the desmoplastic response of CAFs through a paracrine manner 327. While SHH activates PSCs to secrete high levels of perineural invasion (PNI) -related molecules to promote PNI in PC 331. The desmoplastic connective tissue and PNI further maintain the hypoxic environment of the tumor and stabilizes HIF, allowing PC cells to acquire drug resistance.

In the same way, CXCL12/CXCR4 (C-X-C motif chemokine ligand 12/C-X-C motif chemokine receptor 4) signaling axis can confer drug resistance to PC cells and participate in the invasion and metastasis of PC 332, 333. CXCL12 secreted by stromal cells binded to the receptor CXCR4 on PC cells, thereby activating AKT and ERK, leading to nuclear accumulation of NF-kB. As a result, nuclear NF-kB directly binded to the SHH promoter and induces SHH expression 334. When PC cells were treated with gemcitabine, ROS was up-regulated and mediated the nuclear translocation of NF-kB and HIF-1α by activating ERK1/ 2 and AKT. Then NF-kB and HIF-1α bind to the CXCR4 promoter, up-regulate CXCR4 and lead to enhanced motility and invasion of PC cells 335. The adverse reactions of gemcitabine treatment were reflected. First, gemcitabine may enhance the anti-apoptotic pathway downstream of CXCR4, thereby making cancer cells resistant. In addition, gemcitabine may enhance the metastasis of PC cells to other CXCL12 overexpression environments 335.

Lysyl oxidase (LOX) and lysyl oxidase-like protein (LOXL) are copper-dependent amine oxidases that catalyze the covalent cross-linking of collagen and elastin in extracellular matrix (ECM), which are related to the progression of cancer 336-338. There is a hypoxia response element (HRE) on the promoter of human LOX gene to respond to HIF and transcribe LOX protein 339. LOX and LOXL levels in PC cells and PSCs increased with HIF-1α activity 340, 341. LOXL2 can reduce the drug concentration in the tumor during chemotherapy. This may be due to LOXL2 forming a physical barrier to inhibit the diffusion of gemcitabine by increasing fibrous collagen and then increasing ECM stiffness, or it may be due to the collapse of blood vessels in the tumor caused by LOXL2, which can limit the transport of gemcitabine into the tumor interior 342.

Correspondingly, matrix metalloproteinase (MMP) is a kind of proteolytic enzyme, which can decompose ECM protein, promote angiogenesis, and is related to tumor proliferation and metastasis 343, 344. MMP has the opposite effect to LOX, and inhibition of LOX can promote the expression of MMP 345, 346. However, LOX has little effect on the activity of MMP in rat aortic smooth muscle cells 347, and even in gastric cancer 348, cervical cancer 349, colorectal cancer 350, non-small cell lung cancer 351, breast cancer 352, LOX is positively correlated with MMPs or increases MMPs activity, while in liver cancer, lysyl oxidase propeptide (LOX-PP) inhibits liver cancer cell migration by down-regulating MMPs expression 353. The relationship between MMP and LOX remains unclear.

In PC, HIF-1α can promote the transcription of membrane type 2 matrix metalloproteinase (MT2-MMP) 354, 355, and further, MT2-MMP participates in the progression of PC by activating MMP-2^356^. MMP-2 in PC cells and PSC also depends on HIF-1α regulation 340. In addition, HIF-1α binds to the HRE on the fascin promoter and activates its mRNA transcription, and overexpression of fascin can increase MMP-2 expression and promote PC cell migration and invasion 357. Moreover, ROS/MMP-3 signaling pathway is activated by high glucose and up-regulates ribonucleotide reductase catalytic subunit M1 (RRM1) expression, a member of ribonucleoside reductase, inducing gemcitabine resistance in PC 358. MT1-MMP up-regulates the expression of high mobility group A2 (HMGA2), a non-histone DNA-binding nuclear protein involved in chromatin remodeling and gene transcription, to attenuate the therapeutic effect of gemcitabine in PC 359. In addition, the use of MMP-2 inhibitor tissue inhibitor metalloproteinase 2 (TIMP2) and MMP-9 inhibitor TIMP1 increased the inhibitory effect of hyperthermia combined with gemcitabine on the invasion of gemcitabine-resistant PC cells 360.

Vascular endothelial-derived growth factor (VEGF) is another key factor in pancreatic tumor angiogenesis. VEGF plays an important role in the whole process of angiogenesis, and its expression is mainly regulated by HIF 361. In PC, hypoxia can induce the translocation of pyruvate kinase M2 (PKM2) and nuclear factor kappa B subunit 3 (p65, NFKB3) to the nucleus of PC cells, and PKM2 can activate NF-kB to mediate the transcription of HIF-1α and its target gene VEGF 197. In addition, PKM2 is a co-activator of HIF-1α and its downstream target gene 362. VEGF can stimulate PC cells to up-regulate HIF-1α and enhance glycolysis 363. In the PC transplantation model, gemcitabine can reduce the protein levels of VEGF, VEGFR2, platelet and endothelial cell adhesion molecule 1 (CD31, PECAM1) and HIF-1α to reduce blood vessel formation, thereby reducing tumor growth 364. While using other drugs, the small molecule compound LB-100 enhanced the cytotoxicity of doxorubicin by increasing angiogenesis through the HIF-1α/VEGF axis 365. However, inositol tripyrophosphate (ITPP) can down-regulate HIF-1α, VEGF and LOX and improve the sensitivity of gemcitabine treatment, which may be caused by the improvement of vascular structure and the reduction of connective tissue proliferation 366. Metformin can inhibit the mTOR/HIF-1α/VEGF signaling cascade to inhibit the progression of gemcitabine-resistant PC 367. At the same time, metformin can also down-regulate SHH to show the effect of of inhibiting PSCs. Among the downstream effects, the inhibition of VEGF leads to the reduction of neovascularization, the weakening of fibroproliferative reaction, and the enhancement of the anti-tumor effect of gemcitabine 368.

In general, the effect of TME on tumor has two sides, and therapeutic drugs also start from both sides. By reducing angiogenesis and promoting connective tissue proliferation, the cancer cells are kept in a state of hypoxia and malnutrition for a long time, and at the same time, the metastasis of cancer cells is blocked at the physical level to achieve the effect of tumor inhibition. On the contrary, improving the vascular structure and inhibiting the proliferation of connective tissue can make the drug directly reach the inside of the tumor, which is beneficial to the anti-tumor effect of the drug.

## Conclusion

LncRNA can regulate autophagy and Wnt/β-catenin pathway from multiple perspectives. Through the common MRE elements, the information exchange between lncRNA-miRNA-mRNA can be realized. In addition to sponging miRNA, lncRNA can also directly bind to proteins to regulate cell physiological activities. Extensive studies have determined that lncRNAs can regulate intermediate mediators, such as HuR, FOX and HIF, in the process of PC, while regulating autophagy and Wnt/β-catenin pathway, and further regulate chemotherapy resistance of PC cells. Although studies on PC resistance have mainly focused on gemcitabine, these several intermediate mediators also have an impact on the efficacy of other drugs. In PC, not only the differentially expressed lncRNAs may serve as PC markers and targets for therapy, intermediate mediators hold the same potential. Furthermore, lncRNAs show strong specific expression in different tissues and cancers. Whether the specific expression of lncRNA is consistent with the drug resistance pathway of PC remains to be further investigated.

## Figures and Tables

**Figure 4:**
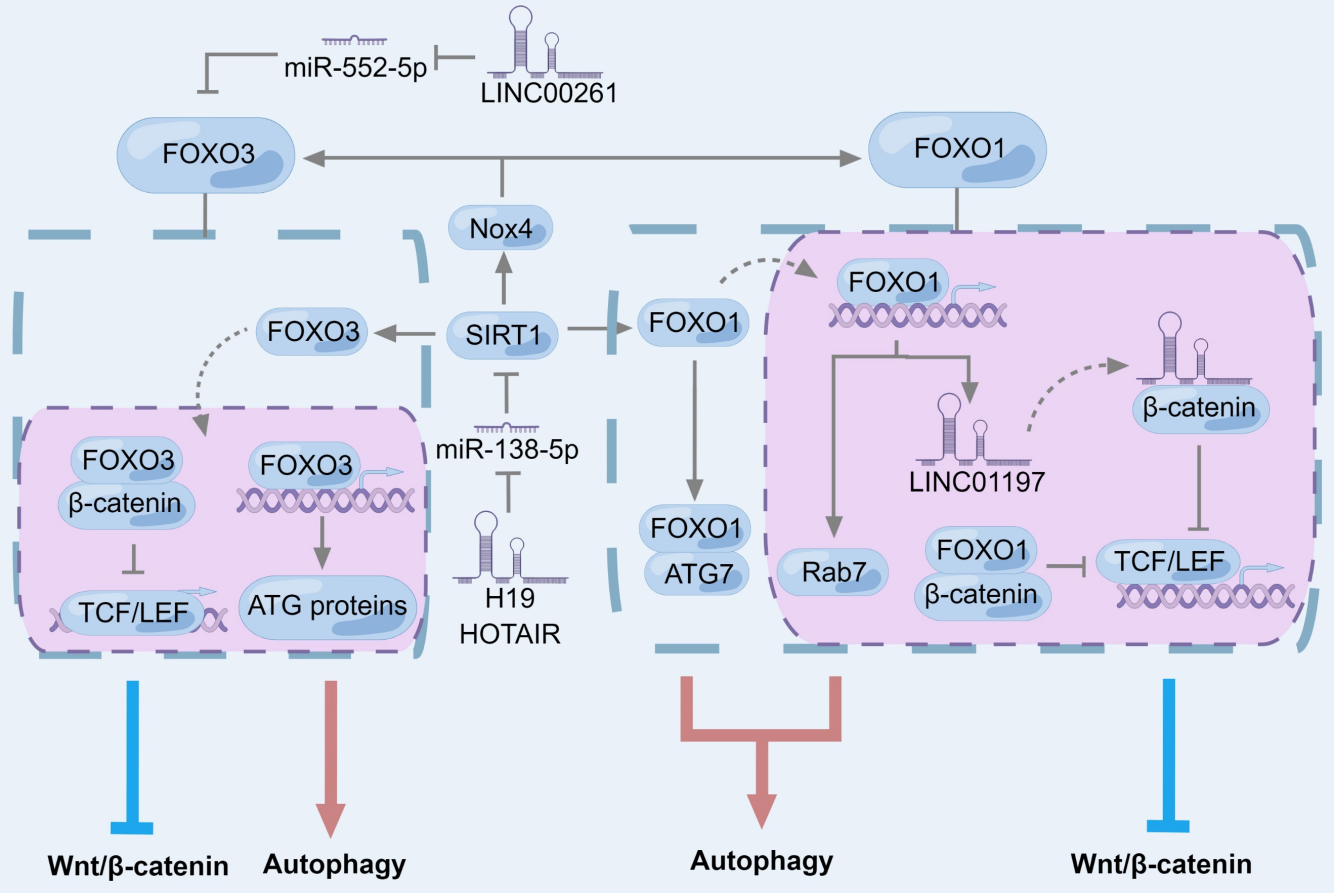
** The role of FOXO1 and FOXO3 in regulating Wnt/β-catenin signaling pathway and autophagy in pancreatic cancer.** Sirt1-mediated deacetylation of FOXO1 and FOXO3 can activate themselves, leading to their nuclear translocation and promoting autophagy. SIRT1 deficiency can induce NF-κB signaling in pancreatic cancer-induced cachexia muscles, enhance Nox4 transcription, and induce FOXO expression. In the nucleus, FOXO1 and FOXO3 bind to β-catenin to inhibit its transcriptional activity. LINC01197, as a target gene of FOXO1, can also bind to β-catenin to inhibit Wnt / β-catenin signaling. In addition to transcription of autophagy-related proteins, acetylated FOXO1 can directly bind to ATG7 to induce autophagy.

**Figure 5:**
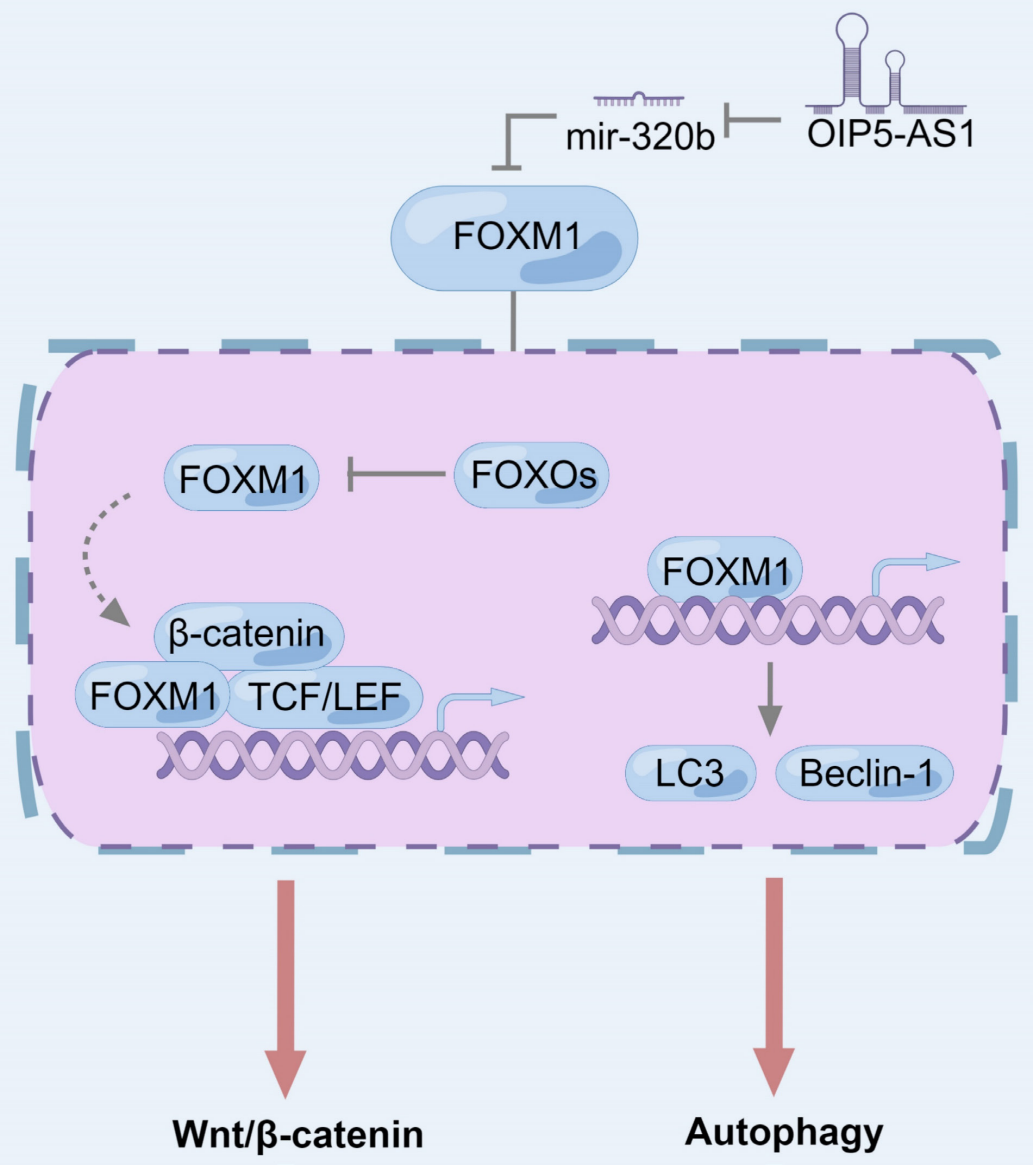
** The role of FOXM1 in regulating Wnt/β-catenin signaling pathway and autophagy in pancreatic cancer.** FOXM1 is inhibited by FOXO subfamily and its interaction with β-catenin can promote the binding of β-catenin to TCF/LEF and activate Wnt/β-catenin signaling. For autophagy, overexpression of FOXM1 and nuclear displacement can regulate the expression of autophagy-related genes, which is manifested as promoting autophagy.

**Figure 6:**
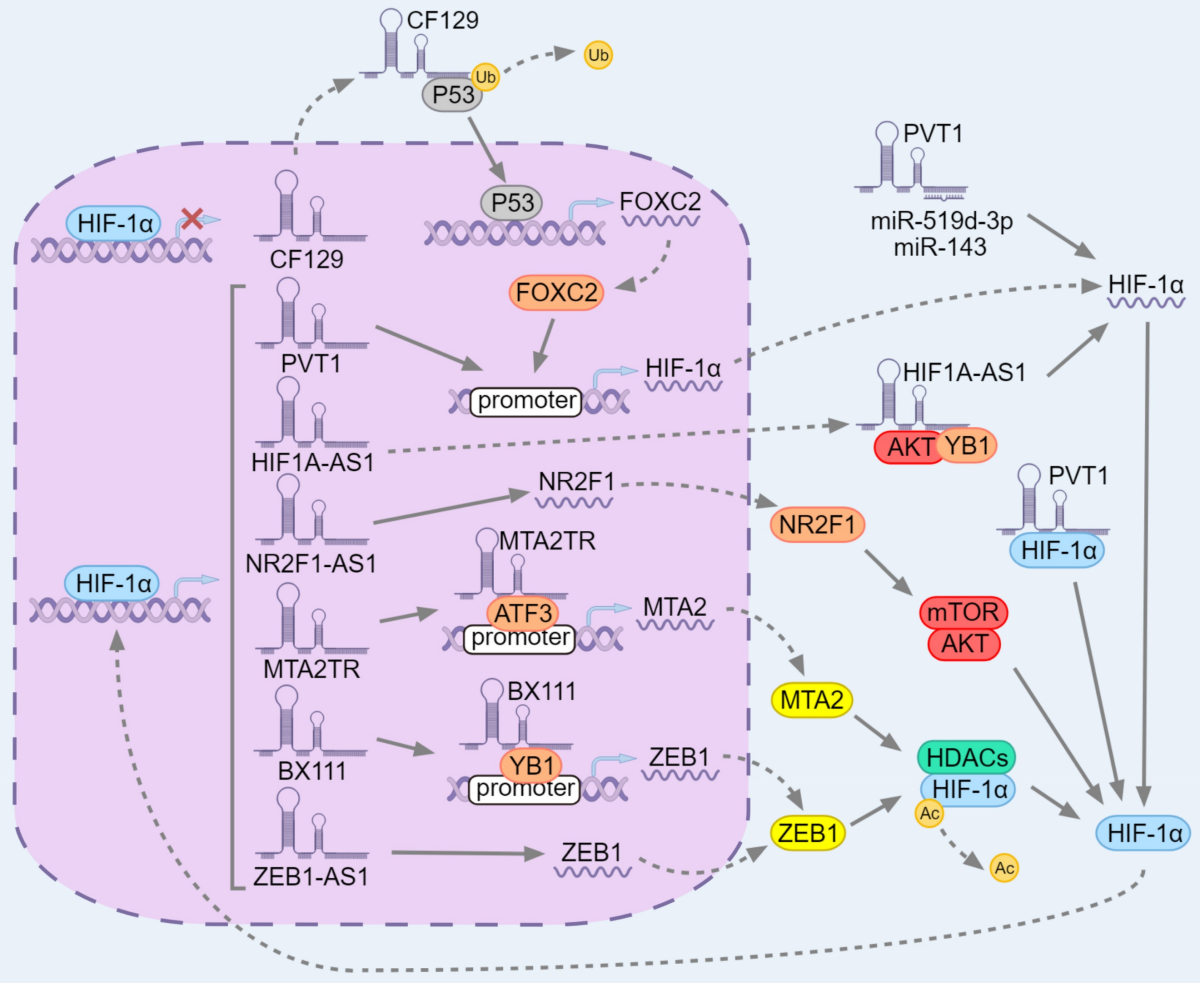
** The positive feedback loop between HIF-1α and lncRNAs in pancreatic cancer.** LncRNAs can be divided into three categories according to the steps of HIF-1α expression. In the HIF-1α transcription stage, HIF-1α can inhibit the transcription of CF129 and then inhibit the ubiquitination of P53. P53 enters the nucleus and transcribe FOXC2, which in turn transcribe the target gene HIF-1α. PVT1 can bind to the HIF-1α promoter and promote the expression of HIF-1α. In the HIF-1α translation stage, HIF1A-AS1 can induce YB1 phosphorylation by AKT and recruit pYB1 to HIF-1αmRNA, thereby promoting HIF-1α translation. PVT1 spongifies miR-519d-3p and miR-143 to stabilize HIF-1αmRNA. After HIF-1α translation, MTA2TR, BX111 and ZEB1-AS1 induce the expression of MTA2 and ZEB1 which in turn cooperate with HDACs to deacetylate HIF-1α. Nr2f1-as1 induces NR2F1 to up-regulate HIF-1α by activating AKT/mTOR signaling. PVT1 interacts with HIF-1α and maintains its protein level.

**Figure 7:**
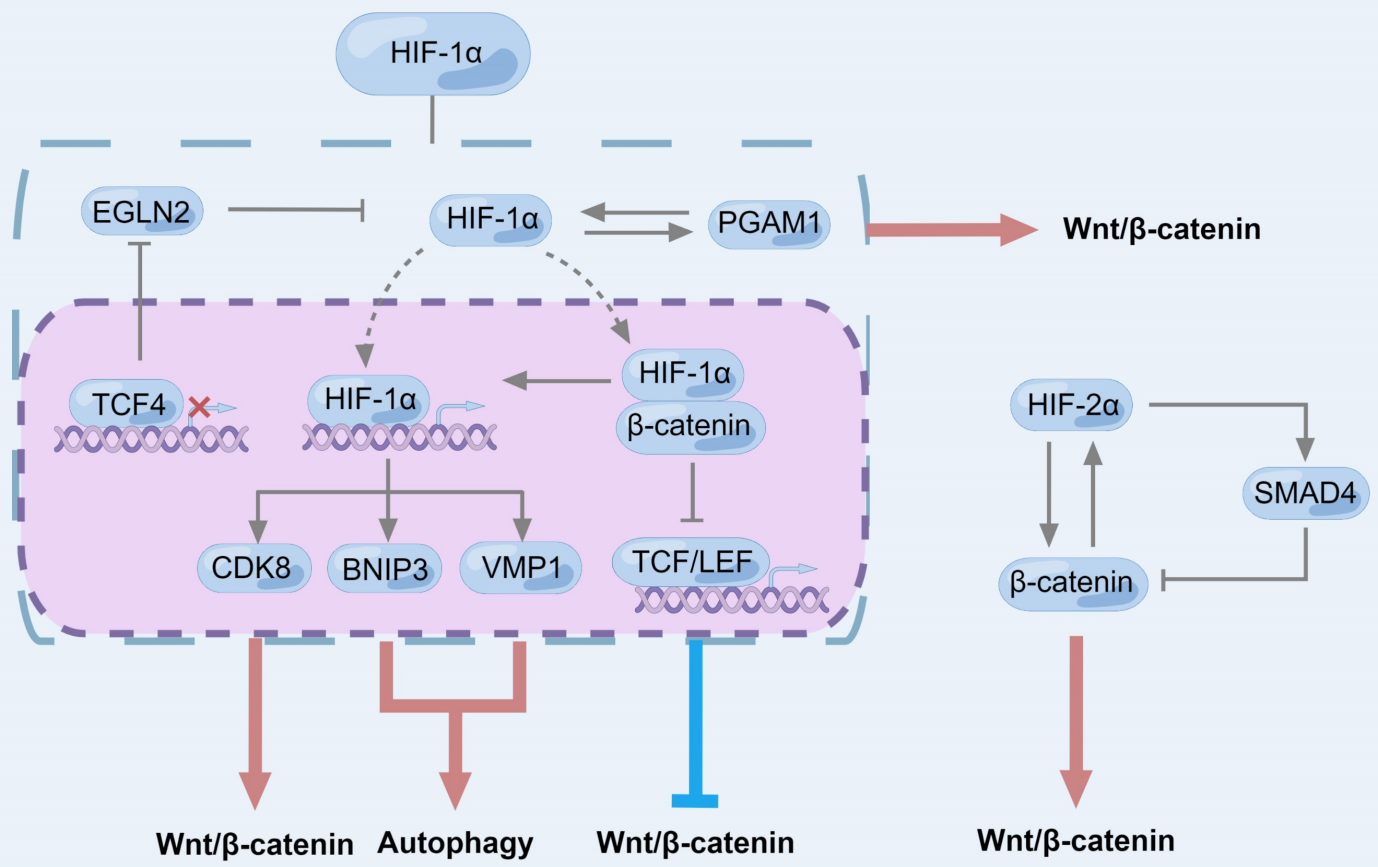
** The role of HIF in regulating Wnt/β-catenin signaling pathway and autophagy in pancreatic cancer.** CDK8, BNIP3 and VMP1 are the downstream factors of HIF-1α, which activate autophagy and Wnt/β-catenin signaling. HIF-1α binds to β-catenin and inhibits the transcriptional activity of β-catenin but up-regulates the activity of HIF-1α. As a β-catenin transcriptional chaperone, TCF4 can inhibit EGLN2 to positively regulate aerobic glycolysis, leading to up-regulation of HIF-1α. PGAM1 mainly exists in cytoplasm and cell membrane and interacts with HIF-1α to positively regulate each other, while up-regulated PGAM1 promotes EMT of pancreatic cancer cells by activating Wnt/β-catenin signaling pathway. Different from HIF-1α, the transcriptional activity of HIF-2α is upregulated after binding to β-catenin. HIF-2α could regulate β-catenin and SMAD4 independently in different ways. There is a competitive relationship between the two pathways, and HIF-2α is more inclined to up-regulate β-catenin to activate the classical wnt signaling pathway in the early progression of pancreatic cancer

**Figure 8:**
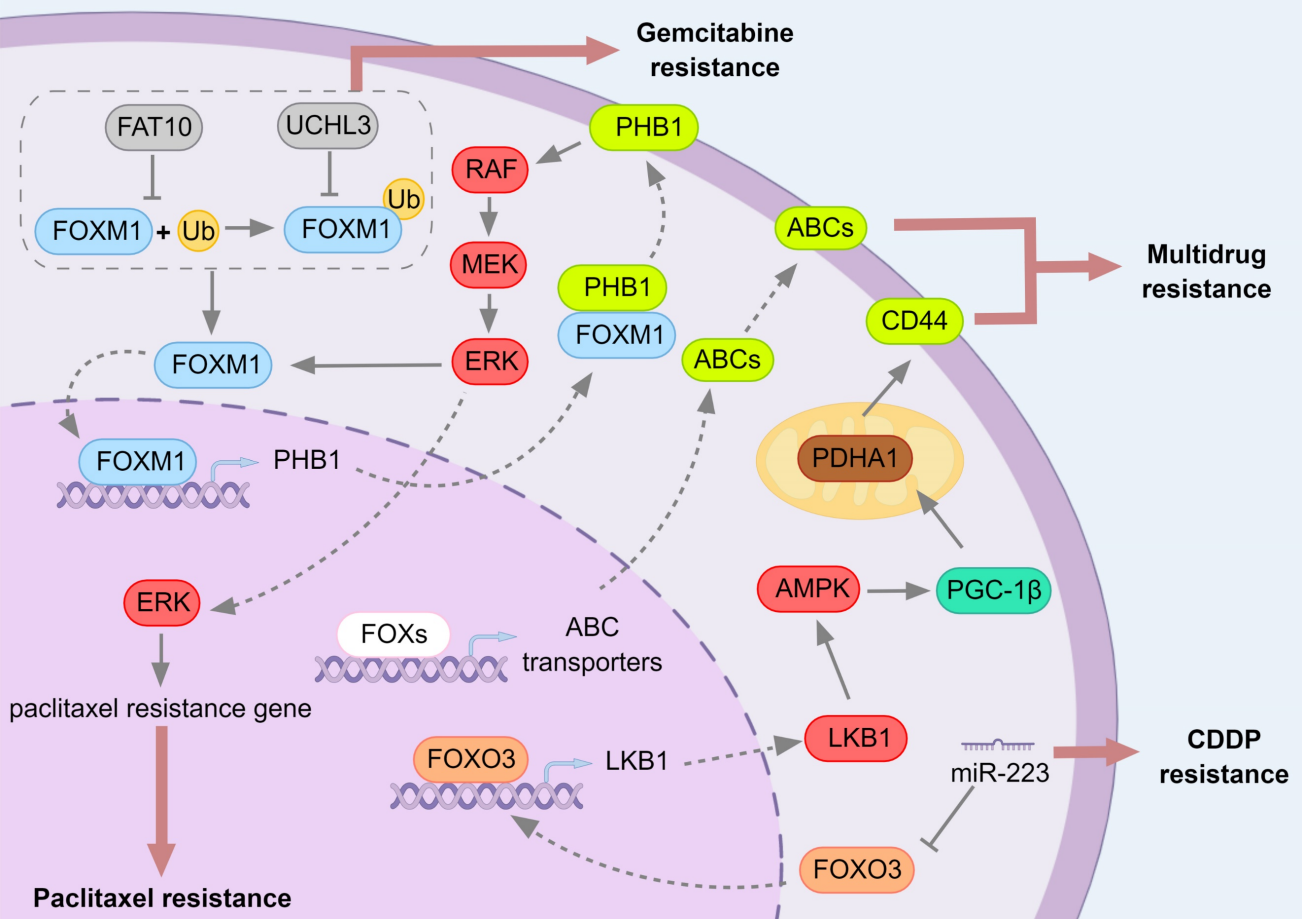
** The role of FOXO3-FOXM1 axis in regulating drug resistance in pancreatic cancer.** A variety of ABC transporters are the target genes of FOXO1, FOXO3 and FOXM1, which can make pancreatic cancer cells resistant to multiple drugs. During FOXM1 deubiquitination, FAT10 competitively binds with ubiquitin to inhibit FOXM1's ubiquitination, while UCHL3 can deubiquitinate the ubiquitinated FOXM1, both of which reduce the sensitivity of pancreatic cancer cells to gemcitabine. PHB1 is a downstream factor of FOXM1, which can activate the RAS-RAF-MEK-ERK pathway to generate a positive feedback loop and lead to the development of paclitaxel resistance. MiR-223 targets FOXO3 to acquire CDDP resistance in cancer cells. cGMP inhibited FOXO3 and affected the CSC phenotype of pancreatic cancer cells through the FOXO3/LKB1/AMPK/PGC-1β/PDHA1/CD44 axis. FOXO3 can maintain CD44 expression and make CSC acquire drug resistance.

**Table 1:** LncRNA regulates PC via Wnt/β-catenin signaling pathway

LcnRNA	Gene and pathway	Interaction with wnt signaling	Cancer phenotype	Reference
BANCR	miR-195-5p	Activate	Promote PC cell proliferation, invasion and migration	129
DGCR5	miR-3163/TOP2A	Activate	Promote PC cell migration, invasion EMT and gemcitabine resistance	369
DLX6-AS1	miR-497-5p/FZD4/FZD6	Activate	Promote PC cell proliferation, invasion, migration and inhibit apoptosis Promote PC growth and metastasis *in vivo*	31
FAM83H-AS1	FAM83H/ β-catenin	Activate	Promote PDAC cell proliferation, migration and invasion *in vitro* and *in vivo*	51
FGD5‑AS1	miR-577/β-catenin, LRP6	Activate	Promote PC cell proliferation, invasion and migration	30
GATA3-AS1	miR-30b-5p/TEX10	Activate	Promote PC cell viability, proliferation, invasion, stemness and inhibit apoptosis	370
H19	miR-194/PFTK1/LRP5/6	Activate	Promote PDAC cell proliferation and migration	34
HOTAIR	WIF1	Activate	Enhance the radiosensitivity of PDAC cells, reduce the proliferation, and increase the apoptosis of cells after radiation	41
HOTAIR	Wnt	Activate	Promote PC cell proliferation, migration, invasion and EMT	371
HOTTIP	WDR5/HOXA9/Wnt	Activate	Enhance CSC properties and promote PDAC tumorigenesis	372
HULC	Wnt	Activate	Promote PC cell proliferation and invasion and inhibit apoptosis	373
LINC01133	EZH2/ H3K27me/AXIN2	Activate	Promote PC cell proliferation, migration, invasion, EMT and inhibit apoptosis	40
LINC01133	DKK1	Activate	Promote PC cell growth, proliferation, migration, metastasis and invasion	39
LINC01614	GSK‑3β/AXIN1	Activate	Promote PC cell proliferation, migration, invasion *in vitro* and tumor proliferation *in vitro* and *in vivo*	35
OIP5-AS1	miR-320b/FOXM1	Activate	Promote PC cell proliferation, migration and invasion	374
PVT1	miR-619-5p/PYGO2	Activate	Promote PC cell viability and gemcitabine resistance *in vitro* and *in vivo*	20
SH3BP5-AS1	miR-139-5p/CTBP1	Activate	Promote PC cell migration, invasion and gemcitabine resistance	375
TSLNC8	HuR/β-catenin	Activate	Promoted PC cell proliferation and invasion *in vitro* and enhance PC growth and metastasis *in vivo*	46
WTAPP1	WTAP	Activate	Promotes PC cell proliferation and invasiveness	49
LINC00261	miR-552-5p/FOXO3	Inhibit	Inhibit PC cell migration, invasion and EMT	157
LINC01197	FOXO1/LINC01197/β-catenin	Inhibit	Inhibit PC cell proliferation and growth	36
MEG3	miR-183/BRI3	Inhibit	Inhibit pNET cell viability, invasion and migration and induce apoptosis.	376
NEN885	Wnt	Inhibit	Inhibit GEP - NEN cell migration, invasion and EMT	377

**Table 2:** LncRNA regulates PC via autophagy

LcnRNA	Gene and pathway	Interaction with autophagy	Cancer phenotype	Reference
HOTAIR	ATG7	Activate	Reduce the radiosensitivity of PC cells	378
Lnc-FSD2-31	miR-4736/ATG7	Activate	Inhibit PC cell growth	67
MALAT1	HuR/TIA1	Activate	Promote PC proliferation and metastasis	92
PVT1	miR-619-5p/ATG14	Activate	Promote PC cell viability and gemcitabine resistance	20
PVT1	miR-143/HIF-1α/VMP1	Activate	Promote PC cell viability and gemcitabine resistance	64
SNHG14	miR-101	Activate	Promote PC cell viability, proliferation and gemcitabine resistance	379
ANRIL	miR-181a/HMGB1	Repress	Promote PC cell proliferation, migration, invasion and gemcitabine resistance	68
LINC01207	miR-143-5p/AGR2	Repress	Promote PC cell growth and inhibit apoptosis	380
LINC01133	miR-216a-5p/TPT1/mTORC1	Repress	Promote PC cell proliferation and metastasis	381
LZTS1-AS1	LZTS1-AS1/miR-532/TWIST1	Repress	Promote PC cell proliferation, migration, invasion and oncogenicity, inhibit apoptosis and autophagy	86
UCA1	UCA1/MAPK/ERK	Repress	Promote PC cell mitochondrial fusion and migration, inhibit mitophagy	89
HCP5	miR-214-3p/HDGF	——	Promote PC-GR cell proliferation, invasion and migration, inhibit cell apoptosis and increase gemcitabine resistance	237

## Abbreviations

5-FU: 5-Fluorouracil
ABC: ATP binding cassette
AGER: advanced glycosylation end-product specific receptor
AGR2: anterior gradient 2, protein disulphide isomerase family member
AMPK: AMP-activated protein kinase
ANRIL: cyclin dependent kinase inhibitor 2B antisense RNA 1
APC: adenomatous polyposis coli
ATF3: activating transcription factor 3
ATG: autophagy related gene
AURKB: aurora kinase B
AXIN: axis inhibitor
BANCR: BRAF-activated non-protein coding RNA
BCL2: B-cell lymphoma 2
BIM: BCL2-interacting mediator of cell death, BCL2L11
BNIP3: BCL2 interacting protein 3
BRI3: brain protein I3
BX111: ZEB1 transcriptional regulator RNA, ZEBTR
CAF: cancer-associated fibroblast
CAR: cardamonin
CCA: cholangiocarcinoma
CCNB1: cyclin B1
CCND1: cyclin D1
CD133+: prominin 1, PROM1 positive
CD31: platelet and endothelial cell adhesion molecule 1, PECAM1
CD44: cluster of differentiation-44
CDK: cyclin dependent kinase
ceRNA: competitive endogenous RNA
CERS6-AS1: ceramide synthase 6 antisense RNA 1
cGMP: cyclic guanosine monophosphate
CK1: casein kinase 1
CNBP: cellular nucleic acid binding protein
CSC: cancer stem cell
CTBP1: C-terminal binding protein 1
CTPS1: CTP synthase 1
CXCL12: C-X-C motif chemokine ligand 12
CXCR4: C-X-C motif chemokine receptor 4
DDIT4-AS1: DNA damage inducible transcript 4 antisense RNA 1
DKK1: dickkopf Wnt signaling pathway inhibitor 1
DLX6-AS1: distal-less homeobox 6 antisense RNA 1
DVL: dishevelled segment polarity protein
ECM: extracellular matrix
EGLN2: egl-9 family hypoxia-inducible factor 2
EIF3B: eukaryotic translation initiation factor 3 subunit B
EMT: epithelial-mesenchymal transition
EVA1A: eva-1 homolog A
EZH2: enhancer of zeste 2 polycomb repressive complex 2 subunit
FAM83H: family with sequence similarity 83 member H
FAT10: human leukocyte antigen F-associated transcript 10
FGD5-AS1: FYVE, RhoGEF, and PH domain containing 5 antisense RNA 1
FOLFIRINOX: combination chemotherapy with fluorouracil, leucovorin, irinotecan, and oxaliplatin
FOX: forkhead box
FZD: Frizzled
GABARAPL1: GABA type A receptor associated protein like 1
GEP-NEN: gastroenteropancreatic neuroendocrine neoplasm
GSK3: glycogen synthase kinase-3
H19: H19 imprinted maternally expressed transcript
H2AX: H2A. X variant histone
H3K27me: methylation of Histone H3 at lysine 27
H3K9ac: acetylation of Histone H3 at lysine 9
HCP5: histocompatibility leukocyte antigen complex P5
HDAC1: histone deacetylase 1
HDGF: heparin binding growth factor
hENT1: human equilibrative nucleoside transporter 1
HIF: hypoxia inducible factor
HMGA2: high mobility group A2
HMGB1: high mobility group box 1
HOTAIR: homeobox transcript antisense intergenic RNA
HOXA9: homeobox A9
HRE: hypoxia response element
HuR: human antigen R
IDH1: isocitrate dehydrogenase (NADP(+)) 1
IPO8: importin 8
ITGB1: integrin subunit beta 1
ITPP: inositol tripyrophosphate
JNK1/2: C-Jun NH2 terminal kinase 1/2
K-RAS: Kirsten rat sarcoma viral oncogene homolog
LC3: microtubule associated protein 1 light chain 3
LEF: lymphoid enhancer-binding factor
LINC: long intergenic non-protein coding RNA
lncRNA: long non coding RNA
LOX: lysyl oxidase
LRP: low density lipoprotein receptor related protein
LZTS1-AS1: leucine zipper tumor suppressor 1 antisense RNA 1
m6A: N6-methyladenosine
MALAT1: metastasis associated lung adenocarcinoma transcript 1
MDR: multidrug resistance
METTL: methyltransferase
MITF: melanocyte inducing transcription factor
MKRN1: makorin ring finger protein 1
MMP: matrix metalloproteinase
MnSOD: manganese superoxide dismutase, SOD2
MRE: miRNA recognition element
MTA2: metastasis associated 1 family member 2
MTA2TR: MTA2 transcriptional regulator RNA
MT2-MMP: Membrane type 2 matrix metalloproteinase
mTOR: mammalian target of rapamycin
MUC1: mucin 1
NF-kB: nuclear transcription factor-kappa B
NNT-AS1: nicotinamide nucleotide transhydrogenase antisense RNA 1
NOX4: NADPH oxidase 4
NR2F1: nuclear receptor subfamily 2, group F, member 1
NSCLC: non-small cell lung cancer
OTUB1: ovarian tumor family deubiquitinase ubiquitin aldehyde binding 1
P53: tumor protein P53, TP53
P62: sequestosome 1, SQSTM1
p65: nuclear factor kappa B subunit 3, NFKB3
PaCSC: pancreatic cancer stem cell
PanIN: pancreatic intraepithelial neoplasia
PARP: poly ADP-ribose polymerase
PC: pancreatic cancer
PDAC: pancreatic ductal adenocarcinoma
PDHA1: pyruvate dehydrogenase E1 subunit alpha 1
PDL1: programmed cell death 1 ligand 1
PGAM1: phosphoglycerate mutase 1
PGC-1β: peroxisome proliferator-activated receptor gamma, coactivator 1 beta
PHB1: prohibitin 1
PIK3C3: phosphatidylinositol 3-kinase catalytic subunit type 3
PKM2: pyruvate kinase M2
PLK1: polo-like kinase 1
pNET: pancreatic neuroendocrine tumor
PNI: perineural invasion
PRC2: polycomb repressive complex 2
PSC: pancreatic stellate cell
PTK7: protein tyrosine kinase 7
PVT1: plasmacytoma variant translocation 1
PYGO2: pygopus family PHD finger 2
Rab7: RAS-related GTP-binding protein 7
RAF1: Raf-1 proto-oncogene
RBP: RNA binding proteins
ROS: reactive oxygen species
RR: ribonucleoside reductase
RRM1: ribonucleotide reductase catalytic subunit M1
SHH: sonic hedgehog ligand
SIRT1: sirtuin 1
SMAD4: drosophila mothers against decapentaplegic protein 4
SNHG16: small nucleolar RNA host gene 16
SP1: specific protein 1
TCA cycle: tricarboxylic acid cycle
TCF: transcription factor
TCF4: T-cell-specific transcription factor 4
TEX10: testis expressed 10
TFE3: transcription factor binding to IGHM enhancer 3
TFEB: transcription factor EB
TIA1: TIA1 cytotoxic granule associated RNA binding protein
TIMP1: tissue inhibitor metalloproteinase 1
TIP60: lysine acetyltransferase 5, KAT5
TKT: transketolase
TLR4: Toll-like receptor 4
TME: tumor microenvironment
TNNC1: troponin C1
TOP2A: DNA topoisomerase II alpha
TPT1: tumor protein, translationally-controlled 1
TRIM22: tripartite motif containing 22
TSC: tuberous sclerosis complex
TSLNC8: tumor suppressive lncRNA on chromosome 8p12
TWIST1: twist family bHLH transcription factor 1
UCA1: urothelial cancer associated 1
UCHL3: ubiquitin C-terminal hydrolase L3
ULK1: Unc-51 like autophagy activating kinase 1
UPF1: upstream frameshift 1
UVRAG: UV radiation resistance associated
VEGF: vascular endothelial-derived growth factor
VMP1: vacuole membrane protein 1
WDR5: WD repeat domain 5
WEE1: WEE1 G2 checkpoint kinase
WIF1: Wnt inhibitory factor 1
WIPI1: WD repeat domain, phosphoinositide interacting 1
WTAP: WT1 associated protein
WTAPP1: WT1 associated protein pseudogene 1
YB1: Y box binding protein 1
ZEB1: Zinc finger E-box binding homeobox 1
ZEB2-AS1: zinc finger E-box binding homeobox 2 antisense RNA 1
